# Systems Biology of the Clock in *Neurospora crassa*

**DOI:** 10.1371/journal.pone.0003105

**Published:** 2008-08-29

**Authors:** Wubei Dong, Xiaojia Tang, Yihai Yu, Roger Nilsen, Rosemary Kim, James Griffith, Jonathan Arnold, H.-Bernd Schüttler

**Affiliations:** 1 Department of Genetics, University of Georgia, Athens, Georgia, United States of America; 2 Department of Physics and Astronomy, University of Georgia, Athens, Georgia, United States of America; 3 College of Agricultural and Environmental Sciences, University of Georgia, Athens, Georgia, United States of America; University of Toronto, Canada

## Abstract

A model-driven discovery process, Computing Life, is used to identify an ensemble of genetic networks that describe the biological clock. A clock mechanism involving the genes *white-collar-1* and *white-collar-2* (*wc-1* and *wc-2*) that encode a transcriptional activator (as well as a blue-light receptor) and an oscillator *frequency* (*frq*) that encodes a cyclin that deactivates the activator is used to guide this discovery process through three cycles of microarray experiments. Central to this discovery process is a new methodology for the rational design of a Maximally Informative Next Experiment (MINE), based on the genetic network ensemble. In each experimentation cycle, the MINE approach is used to select the most informative new experiment in order to mine for *clock-controlled genes*, the outputs of the clock. As much as 25% of the *N. crassa* transcriptome appears to be under clock-control. Clock outputs include genes with products in DNA metabolism, ribosome biogenesis in RNA metabolism, cell cycle, protein metabolism, transport, carbon metabolism, isoprenoid (including carotenoid) biosynthesis, development, and varied signaling processes. Genes under the transcription factor complex WCC ( = WC-1/WC-2) control were resolved into four classes, circadian only (612 genes), light-responsive only (396), both circadian and light-responsive (328), and neither circadian nor light-responsive (987). In each of three cycles of microarray experiments data support that *wc-1* and *wc-2* are auto-regulated by WCC. Among 11,000 *N. crassa* genes a total of 295 genes, including a large fraction of phosphatases/kinases, appear to be under the immediate control of the FRQ oscillator as validated by 4 independent microarray experiments. Ribosomal RNA processing and assembly rather than its transcription appears to be under clock control, suggesting a new mechanism for the post-transcriptional control of *clock-controlled genes*.

## Introduction

**Figure 1:**
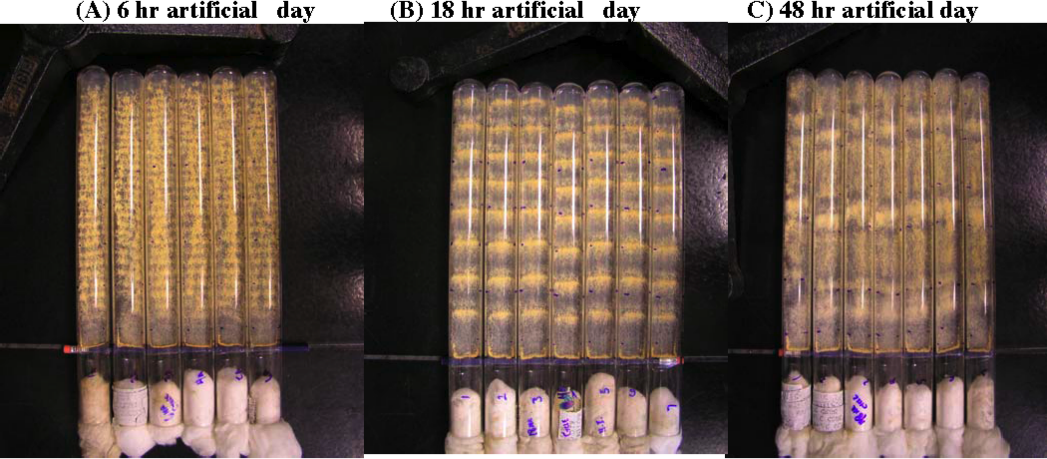
The clock of *N. crassa* is remarkably adaptive in its entrainment to varied artificial days. Replicate race tubes are inoculated at one end and subject to a 6 hr, 18 hr, and 48 hr artificial day over 7 ordinary days. The clock is manifested by the appearance of orange bands (*i.e.*, asexual production of spores) as the culture grows to the other end of the tube. In each artificial day the race tubes experienced (A) 3 hrs light and 3 hrs dark, (B) 9 hrs light and 9 hrs dark, or (C) 24 hrs light and 24 hrs dark. It can be seen that the number of conidial bands tracked the number of artificial days experienced. Race tubes were prepared as described in [30] and in Materials and Methods and were inoculated using the *bd* mutation (FGSC 1858).

To explain how a complex trait works, systems biology begins with organizing macromolecules into a genetic network [1]. The biological clock is an example of how a complex trait with numerous pleiotropic phenotypes can emerge from the interaction of only a few regulatory macromolecules. For two reasons much of what we know about the clock at the molecular level comes from the study of the filamentous fungus, *Neurospora crassa* [2]. First, this complex trait is easy to observe and to manipulate in *N. crassa* (Fig. 1). Second, as a well-studied microbial system, it has been possible to identify three molecular building blocks of the clock, the genes *white-collar-1* (*wc-1*), *white-collar-2* (*wc-2*), and *frequency* (*frq*). The genes *wc-1* and *wc-2* encode PAS-domain containing transcription factors [3] that turn on the clock oscillator. The WC-1 protein also acts as a blue-light receptor [4]. The gene *frq* encodes the clock oscillator FRQ [5] and is activated by the WHITE-COLLAR transcription factor protein complex WCC = WC-1/WC-2. The FRQ protein in turn appears to function as a cyclin to recruit an as yet to be identified kinase/phosphatase pair for the phosphorylation-dependent inactivation of WCC [6].

**Figure 2:**
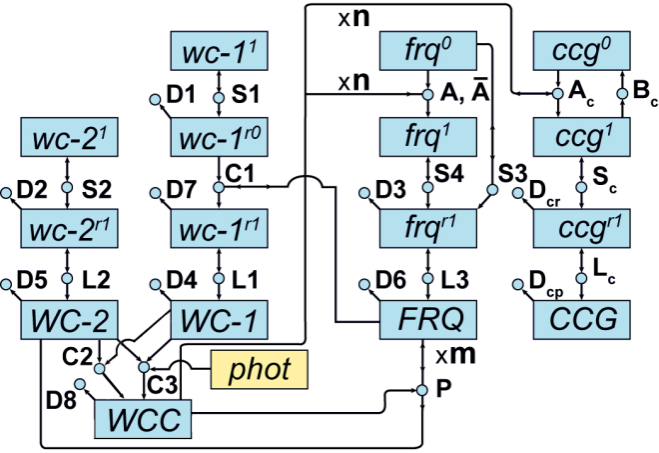
A genetic network for the biological clock from [7]. Molecular species (*i.e.*, reactants or products) in the network are represented by boxes. The *white-collar-1* (*wc-1*), *white-collar-2* (*wc-2*), *frequency* (*frq*), *and clock controlled gene* (*ccg*) gene symbols can be superscripted 0, 1, r0, r1, indicating, respectively, a transcriptionally inactive (0) or active (1) gene or a translationally inactive (r0) or active (r1) mRNA. Associated protein species are denoted by capitals. A phot (in yellow) denotes a photon species. Reactions in the network are represented by circles. Arrows entering circles identify reactants; arrows leaving circles identify products; and bi-directional arrows identify catalysts. The labels on each reaction, such as S_4_, also serve to denote the rate coefficients for each reaction. Reactions labeled with an S, L, or D denote transcription, translation, or degradation reactions, respectively. Reactions without products, such as D_8_, are decay reactions. Reactions, such as A and P, have cooperative kinetics: (A) n WCC+*frq* ^0^→*frq* ^1^ and (P) WCC+m FRQ→WC-2+m FRQ. The n and m are Hill coefficients or cooperativities. Only one reaction, the “A” reaction, has a back reaction, 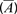, *frq* ^1^→n WCC+*frq* ^0^, included, with non-zero rate. The rate constants specify the right hand side of the kinetics model in equation (1) through the Law of Mass Action in Materials and Methods.

This information enabled formulation of the detailed genetic network shown in Fig. 2 that explains how the clock functions [7]. In this network model, the WCC protein activates the oscillator gene *frq*. The active *frq^1^* gene is then transcribed into its cognate mRNA *frq^r1^*, which in turn is translated into its cognate protein FRQ. The FRQ protein, in turn, deactivates the WCC in the P reaction. FRQ thereby closes a loop of dynamical frustration wherein WCC turns on the oscillator gene whose product shuts down the activator WCC. This dynamical frustration (*i.e.*, negative feedback loop between WCC and FRQ) explains in part how clock oscillations arise [7]. In addition, WCC activates a number of *clock-controlled genes* (*ccg*s) that serve as outputs of this clock mechanism. The number of these *ccg*s in the genome, and hence the extent of clock control over metabolism, is largely unknown but see [8].

**Figure 3:**
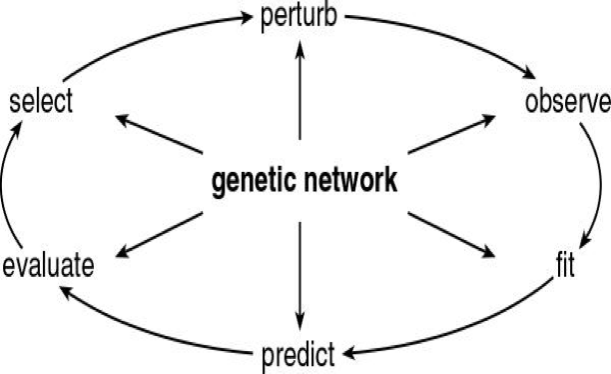
Computing Life Paradigm. The “perturb” and “observe” steps represent the experimentation phase; the “fit”, “predict” and “evaluate” steps are the main components of the genetic network ensemble simulation phase; and the “select” step is the MINE design phase which closes the Computing Life workflow cycle.

Our goal is to refine systematically the genetic network model of the clock mechanism [7] and to explore the metabolic context of the clock. To achieve this goal Locke *et al.* [9] proposed using an iterative process of modeling and experimentation to identify and validate genetic networks. Along these lines, we introduce a model-driven discovery process called Computing Life in Fig. 3 [1], [10]. In this paradigm, a cycle of modeling and genomics experiments are used to identify and, with each cycle, tighten our estimates on model parameters and on model predictions for the biological clock. The biological system is first perturbed. Measurements on all relevant species are made by RNA and protein profiling [1]. An ensemble of genetic network model parameters is generated for the process of interest [11]–[12], [7]. Predictions are made from the model ensemble and compared with available data. Revision of the model then poses the difficult choice of what perturbation experiment is to be done next to improve maximally our knowledge of the genetic network?

One approach to the problem of “informative” experiment design has been to assume that genetic networks are in steady state and/or are linear, and under these conditions predictions are made about the next round of perturbations [13]–[16]. This cannot be done here because the biological clock is usually not in steady state but rather approaching a stable limit cycle [17]. Also, the steady-state approach discards most information contained in observations on network *dynamics*, *i.e.*, its time-dependent behavior. Another approach is to generate an entire compendium of profiling experiments for varied genetic and environmental perturbations [18]. Such profiling experiments are costly, however, and it is desirable that every experiment, at each stage, be maximally informative about the underlying genetic network. Here, we describe how this process of choosing the Maximally Informative Next Experiment (or MINEing) can be guided by the continuously refined network model in an intelligent and cost-effective way while fully exploiting the information contained in the observed network dynamics. Our tracing of three cycles through the Computing Life paradigm in the context of refining our network model for the biological clock's mechanism in *Neurospora crassa* illustrates this approach.

## Materials and Methods

### Describing the genetic network

All stages of the Computing Life paradigm in Fig. 3 involve the use of the genetic network. The methods of describing, fitting, predicting with, and evaluating the genetic network are first described, and then we continue to trace the methodology used to complete the cycle in Fig. 3, providing a methodological walk through the Computing Life paradigm in Materials and Methods.

#### Kinetics model and the model ensemble

The starting point for our MINE design approach is a kinetic rate equation model for the time-dependence of the molecular species concentrations in the network, based, *e.g.*, on mass-action kinetics. The model in Fig. 2, for example, specifies a system of 16 ordinary differential equations (ODEs) that describe the temporal profiles of genes and their products. In general, these ODEs have the form:
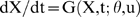
(1)where X≡[X_1_, … X_N_]^T^ is a N×1 vector of species concentrations, with N denoting the number of molecular species evolving according to the kinetics rate equations; G≡[G_1_, … G_N_]^T^ specifies the kinetics, *i.e.*, G_n_(X,t;*θ*,u) is the net rate of production of species n at time t, given the species concentrations X. The model parameter vector or, for the short, “the model” *θ*≡[*θ*_1_, …*θ*_M_]^T^ in G is the M×1 vector of all unknown model parameter variables, including, for example, unknown reaction rate constants, species' initial concentrations and unit conversion factors. The rate functions G also depend on an array of “control variables” which are known and can be varied by the experimenter. These control variables specify, for example, the nature of the perturbations and external conditions to be applied to the biological system or, more generally, the experiment to be done. This array of control variables is denoted by a vector u (of unspecified dimension) and, for short, is referred to as “the experiment” in the following.

### Fitting, Predicting with, and evaluating ensembles of genetic networks

Profiling experiments tend to generate data at only a few time points, the reaction networks are large, and their kinetics models are rich in unknown θ-parameters. An ensemble method of genetic network identification [11], [12], [19], [20], [7] is therefore used to constrain the model parameters θ, using the model likelihood or some other criterion to select members of the model ensemble. The model *ensemble* Q(θ⋅) is a probability distribution on the parameter space of rate coefficients and initial species concentrations [21]. When viewed as a function of θ, the ensemble Q(θ) can be the likelihood function. This model ensemble summarizes what we know and, equally importantly, what we do not know about the biological network, given the prior or “old” experimental data. We refer to Ref. [7] for a detailed description of the construction of Q(θ⋅) from prior experimental data and its numerical implementation by way of a Metropolis Monte Carlo ensemble simulation algorithm *ens.f90*.

With the ensemble in hand it is possible to make predictions from the ensemble means of the species concentrations, as shown in Results. It is also possible to take the expected species trajectories of the ensemble member θ and compare them to the observed species trajectories using a figure of merit, such as the likelihood Q(θ) or χ^2^ = −2 lnQ(θ)+const, to evaluate goodness of fit of the ensemble as described in [11], [7]. A direct graphical evaluation of the ensemble's goodness of fit by can be assessed also by overlaying the observed trajectories of species concentrations onto the ensemble mean trajectories +/− the ensemble standard errors in these mean trajectories, as again shown in Results.

### Selecting an optimal perturbation

The next stage in the Computing Life paradigm is selecting a perturbation in Fig. 3. We describe for the first time a novel method for selecting an optimal perturbation involving evaluating the Maximally Informative Next Experiment.

#### MINE design as “microscopy” in model space

For a given choice of model θ, let f(θ,u) denote a kinetics model prediction for a single species log-concentration, log(y), to be measured for a single time point by the *next* profiling experiment, *i.e.*, y is one of the elements of X, to be measured at some specific observation time t. The vector u, as explained above, comprises all control variables which are known and describe the externally imposed conditions of the experiment. However, u should now be understood *also* to comprise all control variables defining the specific data point y to be measured, including, for example, the choice of molecular species to be observed and the time of observation. We will need to generalize this notation when the planned next experiment measures multiple variables y_1_,…y_d_. Let F(θ, U): = [f(θ, u_1_),…f(θ, u_d_)]^T^ denote d×1 vector of the corresponding predicted log-outcomes and U: = [u_1_,…u_d_] the (super-)vector of corresponding control parameter vectors u_i_ where u_i_ specifies the control variables for the measurement of the data point y_i_ for i = 1, …d. The log-variables to be observed, log(y_i_), will also be referred to, for short, as the “observables” in the following and U, for short, as “the next experiment”. We are using log-concentrations instead of the concentrations themselves here, in order to obtain *scale-free* (*i.e.* concentration-unit-independent) MINE criteria, as explained below.

Clearly, the question of which next experiment U is “maximally informative” is not a mathematically well-defined problem. We have to make an *ad hoc* choice for a design criterion and then try it out in real-life applications. The basic conceptual ideas underlying this *ad hoc* construction of a MINE criterion are borrowed from microscopy: we want to use whatever experimental technique is available to us to “look into” or “image” the inner workings of the cell. A microscope generates images of the cell's material components in three-dimensional *physical space* or in some lower-dimensional projection thereof. Profiling experiments, by analogy, generate images of the cell's (very!) high-dimensional *kinetics parameter space*.

Ideally, we would like to be able to obtain highly resolved images, allowing us to determine accurately a genetic network's “location” in kinetics parameter space, specified by a unique choice of parameter vector θ. Unfortunately, and again in analogy to microscopy, the images we *do* get from present-day profiling experiments do not allow us to completely re-construct θ: our “vision” in θ–space is seriously blurred. The model ensemble Q(θ) captures the constraints imposed on θ, *i.e.*, what we know; but the spread of Q(θ) within those constraints in θ-space, also captures the blurring, *i.e.*, what we do not know, given the *prior* experimental data. Our goal is therefore to reduce this blurring as much as possible when performing the *next* experiment: we want to tune our “model parameter microscope” to get a different view of θ-space with the maximum possible resolution.

**Figure 4:**
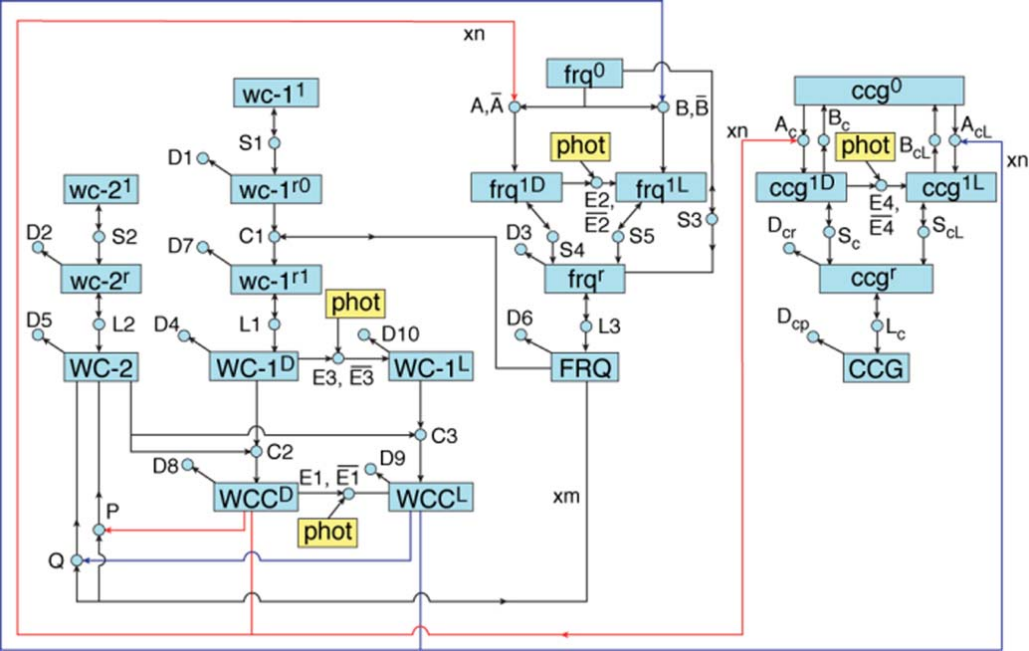
Alternate genetic network for the biological clock from [33]. Molecular species (*i.e.*, reactants or products) in the network are represented by boxes. The terms are the same as described in the legend of Fig. 2. The main difference is that the WCC has a light and dark form denoted WCC^D^ and WCC^L^. When these two forms bind upstream of *frq* and *ccg* genes, this leads to two different transcriptionally active forms of the gene, such as *frq^1D^* and *frq^1L^*. In addition, photons (in yellow) can enter the system to interact with WC-1 in four ways, depending on the bound state of the WCC, in the reactions E1, E2, E3, and E4. All four of these reactions have been given nonzero back reaction rates. The final difference is that the two forms of WCC lead to two deactivation reactions of WCC by FRQ, labeled P and Q. Reactions, such as A and P, have cooperative kinetics: (A) n WCC^D^+*frq* ^0^→*frq* ^1^ and (P) WCC^D^+m FRQ→WC-2+m FRQ. The n and m are Hill coefficients or cooperativities. Only for 6 reactions, such as the “A” reaction, is a back reaction, such as 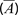 *frq* ^1D^→n WCC+*frq* ^0^, included, with non-zero rate. The rate constants specify the right hand side of the kinetics model in equation (1) through the Law of Mass Action in Materials and Methods.

An important aspect to keep in mind here is that every imaging procedure, be it optical microscopy or RNA profiling, requires a mathematical model which relates the observed image (F) to the underlying object (θ). The mapping function F(θ,U) captures that imaging model. Without such an imaging model, we cannot, for example, re-construct the shape, size and location (θ) of a cellular organelle from the light intensity pattern (F) of the cell's magnified image produced by an optical microscope. In the case of optical and electron microscopes, the imaging model is, by now, well-established, highly reliable, and commonly known as physical optics. In the case of profiling experiments, an appropriate imaging model framework may well be mass balance kinetics, but the details, as illustrated by Fig. 2 or 4 are still very much subject to debate.

Since the profiling experiment is sparse and noisy, we do not have a sufficient amount of sufficiently diverse experimental data to “look in all directions” of the kinetics parameter space. Each experiment only yields a (in general non-linear) projection of object points θ in the M-dimensional kinetics parameter space onto the image points F(θ,U) in d-dimensional image space. Sparsity and noise imply that typically only a lower dimensional image *sub*-space, of dimension d_eff_<M, can actually be resolved by the experiment. The MINE design procedure cannot eliminate the blurring of our vision; but it can help to minimize the blur.

#### Criterion 1: MINE by maximal distance in image space

To develop these notions into a quantitative MINE criterion, let us first consider the simplest case: the design of a Maximally Informative Next Experiment to measure only a single data point y. Suppose we randomly draw two possible choices of models from the model ensemble Q, denoted by θ and θ', which *both* give predictions consistent with the “old” experimental data (within the experimental uncertainties). To distinguish between these two choices, we want to perform the next experiment with control vector u. The predicted outcomes for this next experiment would be, respectively, f(θ⋅,u) and f(θ',u). The crucial point to notice here is this: the more these two predicted outcomes f(θ,u) or f(θ',u) *differ* from each other, the “better” the next experiment will allow us to discriminate between the two model choices. As a “metric” of the difference between the two members of the ensemble, we could choose, for example,
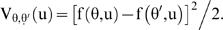
(2)The Maximally Informative Next Experiment u is then the one that maximizes this difference metric. Letting the joint distribution of the randomly drawn pair Q(θ,θ') = Q(θ)Q(θ'), the foregoing criterion can be applied to an ensemble of models by choosing u such that it maximizes the average of V_θ__,θ⋅'_(u):
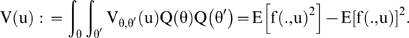
(3)Where ∫_θ_ denotes integration or summation over all θ-components and E[…] denotes the mean over the ensemble probability distribution Q(θ). Our MINE criterion V(u) is then the variance in our prediction within the ensemble and can be evaluated by Monte Carlo (MC) methods [11]. To achieve maximum “resolution” in model (θ-) space, we thus “tune” the next experiment u so that it will take us into those regions of prediction (f-) space where there is the most uncertainty: the MINE design criterion [3] rationalizes and advocates discovery.

A straightforward generalization of the foregoing criterion to the case of the next experiment measuring multiple variables y_1_,…y_d_ is to replace the square of the predicted one-dimensional “image difference”, Δf(θ,θ',u): = f(θ,u)−f(θ',u), in [2] by the corresponding squared “length” of the d-dimensional “image difference vector”
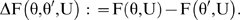
(4)That is, we replace V_θ,θ⋅'_ (u) in [2] by
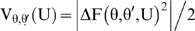
(5)where |…| denotes the Euclidean norm, *i.e.*, |Φ|: = (Φ^T^Φ)^1/2^ for Φ = [Φ_1_,…Φ_d_]^T^. Inserting this into [3] we get the MINE design criterion
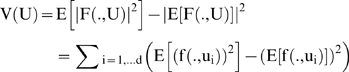
(6)It is easy to see from [6] that maximizing this V(U) will collapse *all* u_i_ at the same u-point where the individual variance in the 1-dimensional (single-variable) prediction space, E[|f(.,u_i_)|^2^]−|E[f(.,u_i_)]|^2^, is largest. So, this MINE criterion would demand that the next experiment simply observe the *same* y-variable d times, instead of observing d independent y-variables. Clearly, this criterion lacks the ability to enforce independence of multiple observables.

#### Criterion 2: MINE by maximal volume in image space

To construct a likely more useful MINE criterion, which *does* enforce some measure of independence of the observables, we are again guided by the microscopy analogue. Suppose we are “viewing” a certain “object space volume” **ν_o_** through our microscope. By way of the mapping function, this produces from **ν_o_** an “image difference volume” **ν**_Δ_ in d-dimensional image difference space. That is, **ν**_Δ_ is the volume swept out by the image difference vector ΔF(θ,θ',U) for all pairs of object points (θ,θ') in **ν_o_**×**ν_o_**; or, formally, **ν**_Δ_(**ν_o_**,U): = ΔF(**ν_o_**,**ν_o_**,U). Our notation makes it explicit here that **ν**_Δ_ depends on the choice of the control vector U, as well as on **ν_o_**.

To formulate an improved MINE criterion, we propose to invoke the volume **ν**_Δ_ swept out by ΔF, instead of the Euclidean norm of ΔF used in [6]. The basic microscopy-inspired idea here is this: the greater the volume amount contained in **ν**_Δ_(**ν_o_**,U), the more detail we should be able to discern in **ν_o_**. In other words, we should be able to gain more information about the contents of **ν_o_** if we tune our microscope's control vector U so as to increase the d-dimensional image difference volume amount, denoted by |**ν**_Δ_(**ν_o_**,U)|. However, unlike the Euclidean distance criterion [6], the requirement of sweeping out a higher-dimensional volume **ν**_Δ_ will naturally enforce a certain degree of independence of the observables. Notice here that the Euclidean norm measures just the length of the ΔF-vector and this can be maximized even if ΔF sweeps out only a 1-dimensional sub-manifold. By contrast, **ν**_Δ_ is by construction a higher-dimensional manifold with a dimensionality of up to d or M, whichever is less.

The next question is then how to choose an appropriate **ν_o_**, or the corresponding **ν**_Δ_, in terms of the ensemble pair distribution Q(θ,θ') = Q(θ)Q(θ'). Again, this requires an *ad hoc* decision and we are guided in making it by computational expediency. In fact, the foregoing considerations of constructing a **ν**_Δ_ from an underlying **ν_o_** in object (θ-) space should only be regarded as a heuristic motivation for introducing such a **ν**_Δ_. As a practical matter, our **ν**_Δ_-based MINE approach is greatly simplified if we do not try to construct an exact **ν**_Δ_ from a given **ν_o_**. Rather, we will define a “representative” **ν**_Δ_, swept out by ΔF(θ,θ',U) when θ and θ' are drawn from “typical” values prescribed by the ensemble pair distribution Q(θ,θ') = Q(θ)Q(θ'). This **ν**_Δ_ will be constructed from the characteristic variance/co-variance ellipsoid of ΔF and it will be again dependent on the control vector U.

To that end, it is conceptually (but not computationally) useful to first define the ensemble distribution of ΔF
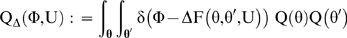
(7)where Φ: = [Φ_1_, …Φ_d_]^T^ is any point in ΔF-space and δ(…) is the Dirac delta-function in d dimensions. Q_Δ_(Φ,U) is the probability density for ΔF(θ,θ',U) to take on the value Φ, given that θ and θ' are independently distributed according to Q(θ) and Q(θ'), respectively. Q_Δ_(Φ,U) defines an effective **ν**_Δ_(U) in the image difference (ΔF-) space by way of the characteristic ellipsoid of ΔF's d×d variance/co-variance matrix D(U), given by
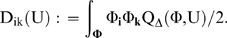
(8)Note that ΔF's characteristic variance/co-variance ellipsoid is centered at the origin, Φ = 0, since Q_Δ_(Φ,U) is even in Φ. *i.e.*, Q_Δ_(−Φ,U) = Q_Δ_(Φ,U) due to ΔF(θ',θ,U) = −ΔF(θ,θ',U). The squared half-axis lengths of the characteristic ellipsoid are the D-matrix eigenvalues, with corresponding eigenvectors defining the respective half-axis orientations. The ellipsoid's half-axes are orthogonal to each other and they define a rectangular prism in ΔF-space whose volume amount, by a universal constant prefactor, is proportional to that of the ellipsoid. Instead of the ellipsoid itself, we therefore choose this “variance/co-variance prism” of ΔF as our image difference volume **ν**_Δ_(U). The square of its volume amount, |**ν**_Δ_(U)|^2^, is the determinant of D(U) and this is what we can use as a possible MINE criterion to be maximized:
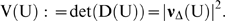
(9)This is sometimes referred to as the *generalized variance*, and its distribution is known exactly if the predictions are Gaussian over the ensemble [22, Th. 3.2.15.]

We should strongly emphasize here that our invocation of the (co-)variance ellipsoid of ΔF does *not* imply or require the ΔF-distribution Q_Δ_(Φ,U) to be Gaussian. *Via* the matrix D(U) in [8], such an ellipsoid can be constructed for any Q_Δ_(Φ,U). Given the *ad hoc* character of the entire MINE approach, the resulting **ν**_Δ_ is “as good as any” for purposes of representing a “characteristic volume” swept out by ΔF(θ,θ',U). The main advantage of this construction is its computational feasibility: combining [4], [7] and [8], we can re-write D_ik_(U) in terms of ensemble means over Q(θ) as
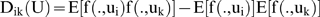
(10)which can be calculated by ensemble Monte Carlo evaluation [7], [11] of the required ensemble means E[…]. We also note here that the D-matrix, as well as the E-matrix defined below, is *scale-free*, that is, independent of the choice of model concentration units, since the definition of D_ik_(U) invokes only ΔF(θ,θ',U) [4] which involves only the *differences* of log-concentrations or, equivalently, logs of only concentration *ratios*. Hence, the D_ik_(U) matrix elements, the corresponding E_ik_(U) matrix elements defined below, and all MINE criteria developed here based on these two matrices are scale-free.

#### Hilbert Space picture of MINE formalism

The foregoing results can also be re-stated in terms of a Hilbert Space (HS) formalism, using a HS of functions defined on the kinetics model parameter (θ-) space where the (co-)variance serves as the HS inner product. That is, for any pair of such functions, g(θ) and h(θ), we define the HS inner product by:
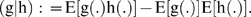
(11)The log variables log(y_i_) are now represented by HS vectors f_i_:
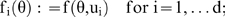
(12)and the (co-)variance matrix element D_ik_ is simply the inner product of HS vectors f_i_ and f_k_,
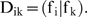
(13)The ensemble standard deviation of the observable predicted by f_i_ is the HS vector norm or “length”, denoted by ∥f_i_∥, where ∥…∥ is defined by ∥g∥: = (g|g)^1/2^. For notational convenience, we are occasionally suppressing the dependence on U or u_i_ for quantities like f_i_ or D_ik_.

Independence of observables log(y_1_),…,log(y_d_) is very naturally represented in this formalism in terms of linear independence of the corresponding HS vector set f_1_,…,f_d_. These d HS vectors span a finite-dimensional subspace in HS which has dimension d, if f_1_,…,f_d_ are linearly independent; else it is less than d. A linearly independent HS vector set f_1_,…,f_d_ also spans a d-dimensional prism in HS, and the MINE criterion [9] has a simple interpretation in terms of this HS prism: from [13], it is easy to show that det(D) is the square of the volume of this HS prism. Hence, the characteristic (co-)variance prism **ν**_Δ_ in d-dimensional ΔF-space has an alternative (“dual”) representation in terms of the HS subspace prism volume. In contrast **ν**_Δ_ in ΔF-space, the HS prism spanned by f_1_,…,f_d_ is, in general, not rectangular since f_1_,…,f_d_ are not guaranteed to be mutually orthogonal with respect to their HS inner product [11].

If the f_1_,…,f_d_ become linearly dependent, their HS prism collapses to a lower-dimensional one, and det(D) vanishes. In the other extreme, if the observables are uncorrelated, the corresponding f_1_,…,f_d_ are mutually orthogonal in terms of the HS inner product, *i.e.*, they are maximally independent: their HS prism volume is simply given by the product of their vector lengths ∥f_i_∥ and hence det(D) = (∥f_1_∥·…·∥f_d_∥)^2^. In general, for correlated observables, the f_1_,…,f_d_ are non-orthogonal and we have det(D)<(∥f_1_∥·…·∥f_d_∥)^2^. The ratio det(D)/(∥f_1_∥·…·∥f_d_∥)^2^ can be regarded as a composite measure of the degree of independence of the observables and this ratio, as discussed further below, depends only on the HS “angles” between pairs of f_i_-vectors, but not on their individual lengths ∥f_i_∥. Maximizing V(U) = det(D(U)) therefore requires a compromise between maximal mutual independence of *all* observables and maximal variance of each individual observable.

We note in passing that the Euclidean distance criterion [6] can also be expressed in terms of the (co-)variance matrix D: the right-hand side of [6] is the trace of D(U). However, in contrast to the volume criterion [9], for the Euclidean distance criterion [6], V(U) is simply the sum of the squared HS vector lengths, *i.e.*, V(U) = trace(D(U)) = ∥f_1_∥^2^+…+∥f_d_∥^2^, and that is maximized when each individual HS vector length is maximal, that is, when each observable has maximal variance, regardless of any co-variance correlations between observables, as already discussed under [6].

#### Criterion 3: MINE by maximal observational independence

Based on these considerations, we propose one further MINE criterion which more strongly than [9] emphasizes independence of the observables. Instead of the original HS vectors f**_i_**, we use normalized HS vectors
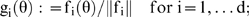
(14)to define a new “normalized” (co-)variance matrix or correlation matrix, denoted by E, analogous to [13],
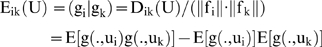
(15)This is the well known correlation matrix between the predictions [22]. Our proposed third MINE criterion is then to maximize
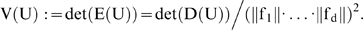
(16)In contrast to [9], the variances of the observables do not affect det(E(U)), only their degree of linear independence does. In HS geometrical language, det(E(U)) is the squared volume of a prism spanned by the HS unit vectors g_1_,…,g_d_, and that volume is determined entirely by the pairwise “angular” relations between the g_i_, not by their individual lengths which are all fixed at ∥g_i_∥ = 1.

Such a MINE criterion is likely advantageous in applications where the HS vectors of the observables, f_1_,…,f_d_, are “almost” linearly dependent. The greatest gain in information from the next experiment is then likely achieved by improving the independence of the observables, rather than by maximizing their individual variances. This scenario of “almost” linearly dependent (*i.e.*, highly correlated) observables is what we have in fact encountered, consistently, in our MINE calculations for the three Computing Life cycles reported in this work. Maximizing det(E(U)) is therefore the MINE criterion we have employed to guide the design of our cycle 1, cycle 2 and cycle 3 experiments, subject to additional numerical modifications now to be discussed.

There are several additional reasons why the det(E(U)) is the preferred MINE criterion. The correlation matrix is a well known measure of linear dependence between variables (*i.e.*, the predictions F) as well as a well known measure of stochastic dependence when the predictions F are Gaussian over the ensemble. The det(E(U)) MINE measure is bounded between 0 and 1. The value of 1 denotes linear independence, and in the case of Gaussian predictions, complete stochastic independence of the predictions. The value 0 means perfect linear dependence of the predictions, and in the case of Gaussian predictions, perfect stochastic dependence. The measure is familiar and easy to interpret, and finally, the det(E(U)) has well known distributional properties, particularly when the predictions are Gaussian [22].

#### Volume collapse pathology

The lack of sufficient linear independence of the g_1_,…,g_d_ HS vector set (or, equivalently, of the f_1_,…,f_d_ set) is most easily diagnosed numerically by calculating the d eigenvalues of the D-matrix, denoted by λ_n_ = λ_n_(U) and enumerated by n = 1,…d in descending order, with corresponding complete, orthonormal d×1 eigenvectors e^(n)^. Since D is non-negative, so are the exact λ_n_. Given the exact λ_n_ and e^(n)^, we can decompose D into its eigenvector representation
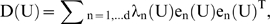
(17)and det(D) is simply the product of the exact λ_n_. However, in our actual MINE calculations, we encounter the numerical difficulty that the HS vector set is numerically “almost” linearly dependent. This numerical pathology manifests itself in the fact that the ratio of smallest eigenvalue λ_d_ to largest eigenvalue λ_1_ becomes of order or smaller than the machine precision ε_mp_. All eigenvalues λ_n_ for which the numerical λ_n_/λ_1_–ratio is comparable to or less than ε_mp_ are then dominated by rounding errors, *i.e.*, they are numerically not calculable and neither, therefore, is det(D). In geometrical terms this simply means that, in the D-matrix characteristic ellipsoid, the ellipsoid half-axis along the corresponding eigenvector direction e^(n)^ has “almost” collapsed to zero and is numerically indistinguishable from zero, √(λ_n_) being the length of that half-axis. However, such an almost collapsed ellipsoid still contains useful information about the “range” swept out by the image-difference function ΔF which can be exploited for MINE design.

To remedy the “ellipsoid volume collapse” pathology, we therefore propose to introduce a numerically stable lower cut-off into the eigenvalue spectrum of D, by replacing λ_n_ with a modified eigenvalue μ_n_ according to
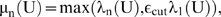
(18)with a fixed “cut-off ratio” ε_cut_ = 10^−10^. This is typically at least 2 or 3 orders of magnitude larger than the machine precision ε_mp_. An almost collapsed characteristic ellipsoid is thus “fattened up” to have a half-axis of at least √(ε_cut_λ_1_) along every eigenvector direction. The numerically inaccessible exact det(D) is then replaced by the numerically stable determinant of a modified D-matrix,
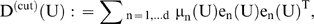
(19)with
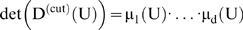
(20)for purposes of MINE calculations. To use the MINE criterion [16] instead of [9], the same cut-off procedure can be employed to generate a modified, numerically stable E-matrix with determinant det(E^(cut)^(U)). This is what we have actually done in the MINE calculations reported here. We also note in closing that the Euclidean distance criterion [6], V(U) = trace(D(U)), is numerically not affected by the ellipsoid volume collapse: trace(D) is the sum, not the product, of the D-eigenvalues and it is therefore dominated by the numerically well-controlled largest eigenvalues only.

#### Cycle 1–3 kinetics ensemble simulations and MINE calculations

In all MINE calculations reported here, the observables log(y_1_),…log(y_d_) were chosen to be the log-concentrations of the 3 clock RNA species which are represented in our network model shown in Fig. 4: log([*frq^r^*]), log([*wc-1^r^*]), and log([*wc-2^r^*]) where [*wc-1^r^*] is the combined total of the “*r0*” and “*r1*” versions of the *wc-1*-RNA, *i.e.*, [*wc-1^r^*]: = [*wc-1^r0^*]+[*wc-1^r1^*]. Each of these 3 RNA concentrations was to be measured at 13 observation time points, t_j_ with j = 1,…13; hence there were d = 3×13 = 39 data points y_1_,…y_d_ to be observed in the next experiment and the index “i” in the above sections therefore represents both the time index j and the species index n, *i.e.*, i↔(j,n) with n = 1,2,3 for the 3 clock RNA species. The observation times t_j_ are chosen to have equidistant spacing t_S_, after an initial time lag of t_L_, measured from the starting time of the experiment, t = 0, where t = 0 is defined by the initial Light-to-Dark (L/D) transition. Hence,
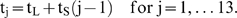
(21)


In each MINE cycle, an ensemble simulation was performed for the kinetics model for Fig. 4, using the same ensemble simulation procedure as described in [7], to generate a “representative” MC sample of 40,000 random θ-vectors, drawn from the respective ensemble distribution Q(θ) for that cycle. A subset of 200 random θ-vectors from this sample was then used to calculate MC estimates for the ensemble expectation values E[…] for evaluation of the E-matrix *via* [15].

In the cycle 1 ensemble simulation, Q(θ) was constructed, as described in [7], from the same prior (“old”) experimental input data as shown in Figs. 2 and 4 of Ref. [7], taken from the literature, Refs. [3], [23], [24] and [25]. For the cycle 2 (cycle 3) ensemble simulation, Q(θ) was revised by adding the new experimental data, from the new cycle 1 (cycle 2) experiment to the cycle 1 (cycle 2) prior experimental data set. In addition, the cycle 2 and cycle 3 ensemble simulations included conidiation density data from the 48 hr artificial day, interpreted as a measure of the CCG protein concentration in Fig. 1, as described in [7]. These conidiation data were generated in a race tube experiment with a periodic light/dark (L/D) exposure with a 48 h period and are the data shown in the right-most panel of Fig. 1.

In all *new* experiments reported here, where light exposure was applied, the light intensity at the sample location was about 70 µmole(photons)/(s·m^2^) in Einsteinian units, or about 15 W/m^2^ in radiometric units, or about 5,300lux∼490 ft-candles in photometric units, assuming a “cool white” spectral distribution, generated by our fluorescent light source. [The approximate conversion factors are 1 W/m^2^↔4.622 µmole(photons)/(s⋅m^2^)↔350.7lux = 32.58 ft-candles for the “cool white” spectrum, as given in [26].] By contrast, the light intensity was only 20 µmole(photons)/(s⋅m^2^), with unspecified spectral distribution, for the light exposure experimental data we have taken from the literature [25]. Since our kinetics ensemble simulations for cycle 2 and 3 incorporate both our new experimental data and the literature data [25] into the respective distributions Q(θ), we have assumed that both, the literature experiments and ours, were performed with the *same* photon spectral distribution. We have therefore modeled all light exposure experiments in terms of photon “concentrations” [entering into the reaction rate function G in [1]] which are chosen *proportional* to the respective light intensities. For all light-exposed experimental data used [25] or reported here, the light exposure was periodic, starting at the initial (t = 0) L/D transition, with a 50% duty cycle (*i.e.*, the same duration of D and L) and a phasing of either D/L (*i.e.* dark first, then light) or L/D, as indicated in the discussion of the respective results. This time-dependent light exposure was modeled, as in [7], by a corresponding time-dependent periodic photon concentration of rectangular pulse shape, entering into in the rate function G.

**Figure 5:**
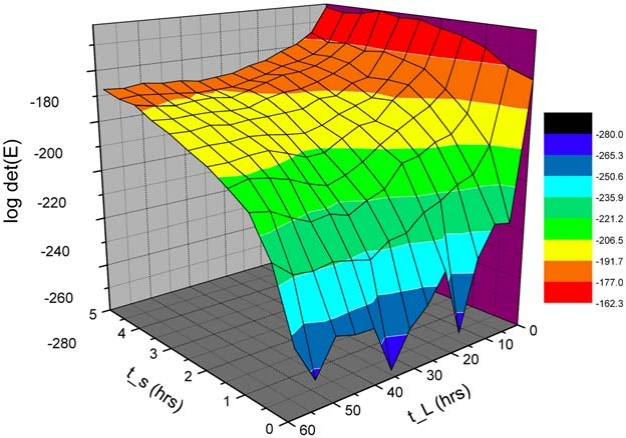
MINE calculation to determine when to start sampling (t_L_) and how often (t_S_). The MINE surface is plotted as function of the lag t_L_ in hrs and spacing t_S_ in hrs; higher values on the MINE surface suggest the preferred design points (t_L_, t_S_). Color contours of the log of the MINE criterion det(E) are overlayed as a function of the lag (t_L_) and spacing (t_S_) to show points on the surface of similar MINE values. The MINE surface suggests to start sampling immediately (small t_L_) and to make the spacing (t_S_) between observations as large as possible. The maximum permissible spacing (t_s_) between observations is 5 hrs, as determined by two constraints. One, there is the cost constraint of 13 microaray chips per cycle, and two, beyond a 50 hr experiment in cycle 1 stable oscillations in liquid culture are not guaranteed.

In cycle 1, V(U) = det(E(U)) was maximized with respect to t_S_ and t_L_, for a “next” experiment designed to measure the three clock RNA species in the dark. The resulting optimal MINE values of t_S_ = 0 and t_L_ = 5 h (see Fig. 5) were slightly modified, to t_S_ = 0 and t_L_ = 4 h, so as to keep the total duration of the cycle 1 experiment below the ∼50 h limit imposed by experimental clock stability constraints in liquid cultures. The latter values of t_S_ and t_L_ were then used, without further adjustments, in all subsequent cycle 1, cycle 2, and cycle 3 RNA profiling experiments and in the corresponding cycle 2 and cycle 3 MINE calculations.

In cycle 2, V(U) = det(E(U)) was maximized with respect to the period t_P_ of the alternating light exposure, for a “next” experiment designed to measure the three clock RNA species subject to a 50% duty cycle and D/L phasing. From the resulting optimal range of t_P_ ∼20 h–24 h (see Results), t_P_ = 24 h was chosen for the actual cycle 2 experiment.

In cycle 3, V(U) = det(E(U)) was maximized with respect to the “gene knock-down” transcription ratio (TR), and with respect to the choice of the gene species to be knocked down, for a “next” experiment designed to measure the three clock RNA species in the dark, but with one of the three clock genes subjected to an experimentally controlled reduction in its transcription rate coefficient. The resulting “most informative” gene was found to be *wc-1*, with a transcription rate coefficient reduced to TR = 10% of the wild-type value (see Results). In the actual cycle 3 experiment, this MINE-recommended knock-down value for *wc-1* was approximated, within the limitations of experimental control, by a TR of about 30% of the wild-type value. The clock RNA data from this *wc-1* knock-down experiment were then incorporated with all other prior (literature, cycle 1 and cycle 2) experimental data into the Q(θ) for one “terminal” ensemble simulation for the network in Fig. 2 and 4. The clock RNA results from this terminal ensemble simulation, along with the respective new RNA data generated in the 3 MINE cycles, are shown in Results for the network in Fig. 4.

### Perturbing the genetic network

Once the MINE perturbation experiment is designed, the perturbation is implemented at the next stage in the cycle in Fig. 3., as now described.

#### Strains

All but one strain used (namely OR-74A below) carry a *band* (*bd*) mutation, permitting the observation of the clock in race tubes. The *bd* mutation (Fungal Genetics Stock Center 1858) was used for the first series of microarray experiments in the dark as well as the second series of microarray experiments examining a light-response. Strain 87-84-6-8 [7] carrying a mutation in *wc-1* and an inducible copy *qa:wc-1^+^* at the *his-3* locus was used in the dial down of *wc-1* expression [7]. Dial-down was achieved by shifting liquid cultures from 0.3% quinic acid to 2% galactose. Strain 74A-OR23-1A was used in shift experiments from sucrose (1.5%) to quinic acid (.3%) as a control to identify QA inducible genes [27]. Strain 93-4 (*frq* ^−^) *qa:frq^+^* transformed with pDE3dBH*qa:frq^+^* was kindly provided by Deborah Bell-Pedersen (Biology Department, Texas A & M University) to test for auto-feedback loops in *wc-1* and *wc-2*.

#### Liquid Growth Conditions for harvesting RNAs

Establishment of liquid cultures followed Nakashima [28] and [5], and the cultures were grown for 48 hrs in petri plates. Half-cm mycelial disks were cut from the mat and dropped into 500 ml flasks with 100 ml Fries+2.0% glucose+.5% arginine+supplements [29].

#### Cycle 1

In the first series of microarray experiments to identify circadian genes (cycle 1) all flasks were placed in a shaker (New Brunswick Scientific, Edison, NJ, Series 25) at 150 rpm and at 25°C for the same period of time, 50 hrs, and also were given at least 2 hrs of 70 micromoles per second per meter squared (µM/s/m^2^) before L/D (light to dark) transition. A total of 13 flasks were harvested by vacuum filtration, one flask every 4 hrs starting at time 0, the L/D transition time, in such a way that the total growth time of each liquid culture was kept constant [42]. After the L/D transition initiating the experiment, the flasks were shaken at 150 rpm and left in the dark. Cells were placed at −70°C to await RNA harvesting using a High Pure RNA kit (Roche, Inc.). The harvested RNA was subjected to RT-PCR and then microarray analysis as described below.

#### Cycle 2

In the second series of microarray experiments to study the light-response (cycle 2) the total growth time was not controlled, and 24 hrs after the L/D transition the light (70 µM/s/m^2^) was turned back on for 24 hrs. 17 flasks were harvested at time points 0 hr, 4 hrs, 8 hrs, 12 hrs, 16 hrs, 20 hrs, 24 hrs, 24 hrs+20 m, 24 hrs+40 m, 25 hrs, 26 hrs, 28 hrs, 32 hrs, 36 hrs, 40 hrs, 44 hrs, and 48 hrs.

#### Cycle 3

In the third series of microarray experiments to study the WCC-response (cycle 3), prior to the L/D transition, mycelial disks were transferred into 500 ml flasks with 100 ml Fries+0.3% quinic acid [29]. Fourteen flasks received a total of 4 hrs of 70 µM/s/m^2^ before L/D transition. Cultures were transferred by vacuum filtration to new 500 ml flasks with 2% galactose+Fries medium and placed in the dark. Flasks were harvested at 0, 10 m, 20 m, 30 m, 40 m, 50 m, 1 hr, 2 hrs, 4 hrs, and 8 hrs. Four additional replicate 0 time points were harvested as well.

### Observing the outcome of the perturbation

Observing the outcome of the perturbation completes the cycle through Fig. 3. Measurements are made on the system by a combination of race tube assays, real-time polymerase chain reaction (RT-PCR), and profiling with oliginucleotide arrays, as now described.

#### Race tube assay for biological clock

Starter cultures were made on 0.1% glucose+0.17% arginine+Vogel's medium [29] and subject to a 23 µM/s/m^2^ light source. Conidia were filtered with glass wool as described in [30] and used to inoculate race tubes layered with 20 ml of 0.1% Glucose+0.17% arginine+Vogel's medium or 0.001 M quinic acid+0.17% arginine+Vogel's medium [29]. A total of 135 replicates tubes were inoculated and either subject to low (23 µM/s/m^2^) or high intensity pulses (70 µM/s/m^2^) once per hour for 90 sec over a twelve hour period or subjected to no light pulses to measure period and a phase response as described in [30] and [31].

#### RNA isolation

RNAs were isolated using the High Pure RNA isolation kit (Roche, Inc.). Their quality and quantify was assessed using an RNA Nano LabChip (Agilent Technologies, Inc.). Generally only samples with a ratio of at least 1.00 for 28S/18S rRNA on the LabChips were used.

#### Real-time PCR to validate microarray experiments

Results on Combimatrix chips were cross-validated by *real-time polymerase chain reaction* (RT-PCR). cDNAs were synthesized from 1.6 µg total RNA using a High-Capacity cDNA Archive (synthesis) kit (Applied Biosystems, Inc.). The *wc-1*, *wc-2*, *frq*, *kal-1*, *rpn-4*, *rrg-1*, *pab-1*, *rok-1*, *lhp-1*, and *rRNA* cDNAs were detected by RT-PCR (ABI-Prism 7500, Applied Biosystems, Inc.) according to manufacturer directions using *Taq* Man probes against an rRNA standard. Triplicate reactions (50 µl) were analyzed using the ΔΔC. method (Applied Biosystems, Inc.).

#### Design of 12K Oligonucleotide Arrays (Combimatrix, Inc.)

**Figure 6:**
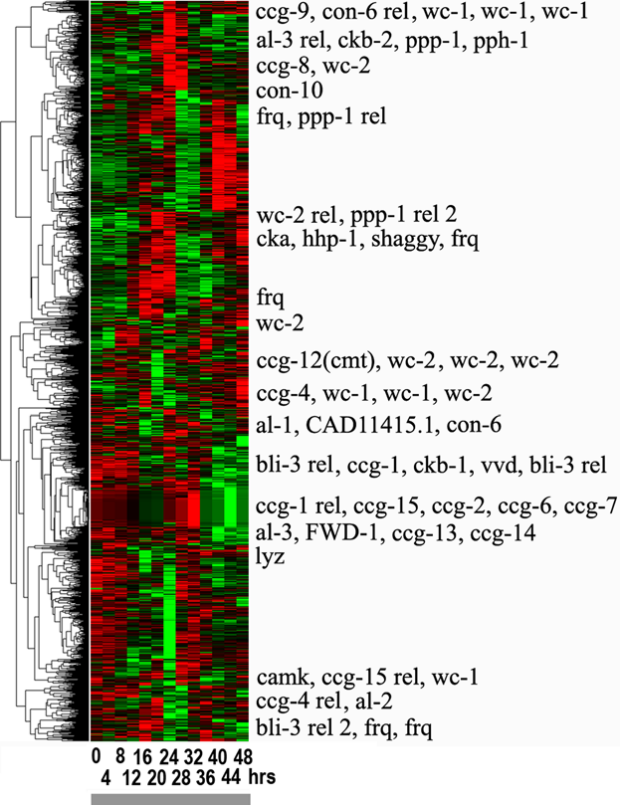
Transcriptional profile of approximately 2436 putative genes with LREs upstream at 0, 4, 8, …, 48 hrs (values staggered on x-axis) after shift from light to dark (L/D) after background subtraction, normalization within arrays relative to the grand median of each chip, logging, and clustering with average linkage using Euclidean distance between mRNA profiles of different genes [38]. The bright green is −3, and the bright red is +3 is expression level on a decadic log scale. Data arose from 13 chips probed with a biotin labeled aRNA. Over 43 known clock-associated genes are overlayed in the right margin of this microarray experiment including varied known *ccg* genes. Genes in Fig. 2 are represented at least 5 times on each chip (explaining why *frq* appears 5 times in the margin). The 2436 putative genes with LREs upstream were selected by fitting A_0_+A sin (ωt+φ) by nonlinear least squares [44] to the profile of each of the 11,000 genes, and those with a significant regression sum of squares contribution with respect to the amplitude A in F_1,9_>5.12 (α = 0.05) [94] and with a period between 16 hrs and 30 hrs are displayed (see Fig. 1 in [5] for *frq* ^r^ mRNA peak separation of 16 hrs and 30 hrs). This work [5] establishes a standard for what is considered acceptable variation in the estimated period of the oscillator. See text for a reexamination of this standard. The smallest significant F_1,9_ ( = 5.12) observed among circadian genes with upstream LREs had an estimated amplitude of 497 and 3.98 fold variation in mRNA levels over time in contrast to the amplitude of *frq* mRNA, 974+/−79. The smallest significant amplitude (63 with an F_1,9_ = 8.29) estimated among circadian genes with LREs upstream had 1.44 fold variation in mRNA levels over time. A gray bar at the bottom indicates lights off.

These arrays were constructed with Version 3 of the *Neurospora crassa* genome sequence [32] from a file labeled neurospora_3_gene_dna_3205.txt downloaded from the Broad Institute Web site. From this sequence Combimatrix, Inc. designed 12,000 oligonucleotides of ∼35 nt to synthesize electrochemically on their chips. Several genes were represented multiple times with oligonucleotides derived from the following genes: *wc-1* (5 duplicates); *wc-2* (5); *frq* (5); *rDNA* (>7), *qa-x* (5); *qa-2* (5), *qa-4* (5); *qa-3* (5); *qa-y* (5); *qa-y* (5); *qa-1F* (5); *qa-1S* (5). In addition, 633 negative control oligonucleotides were added from bacterial, plant, and λ-phage sequences as well as features with no oligonucleotides and quality control oligonucleotides. The rDNA derived oligonucleotide sequences were treated as negative controls in the microarray experiments as well because none of the at least eight rDNA-derived oligonucleotides on each chip showed up in an expanded Fig. 6 with 4721 circadian genes [33] with or without a known LRE upstream. In addition 22 duplicate oligonucleotides from each of 4 distinct λ-phage sequences were added to be used as positive controls for all but 4 arrays (by spiking them into the amplified RNA (aRNA) probe). These positive and negative controls were scattered at random on each array. The arrays with their design are at http://www.yale.edu/townsend/Links/ffdatabase/introduction.htm [34]. Their accession numbers are 13, 34, and 36 for cycle 1, cycle 2, and cycle 3, respectively. The same design was used on all Combimatrix chips except for samples 48 hrs, 44 hrs, 40 hrs, and 36 hrs on cycle 1.

#### RNA amplification and oligonucleotide array hybridization

750 ng of total RNA (as determined by a Nano LabChip (Agilent Technologies, Inc.)) was subjected to one round amplification using the MessageAmp aRNA Amplification Kit (Ambion Inc), which uses an “Eberwine type” amplification. Biotin-11-UTP and CTP (Enzo Life Sciences, Inc.) were incorporated during the *in vitro* transcription reaction. A total of 5 µg of amplified RNA (aRNA) was fragmented, A total of 10 pM each biotinylated spike-in oligonucleotide (phage) was added with hybridization solution, and hybridized according to manufacturer's protocol rev 2.03 (http://www.combimatrix.com). Hybridization was performed at 45°C for 24 hrs using a 25% formamide based solution. Washing was done according to manufacturers protocol rev 2.03. Streptavidin Alexa Fluor® 647 conjugate (Invitrogen) was used at a final concentration of 1.0 µg/ml to visualize hybridized targets. Laser confocal scanning was performed on a GSI Lumonics ScanArray 5000 (now manufactured by Perkin-Elmer, Inc.) using a single laser power and a photomultiplier (PMT) gain setting adjusted less than 10% between arrays. Versions of image software MI_Version_5_4_3, MI_Version_5_5_0, MI_Version_5_6_0, and MI_Version_5_7_0 (Combimatrix, Inc.) were used to obtain spot intensities (such as median foreground count) on each array feature for microarray analysis.

#### Quality Control on RNAs

RNA samples was confirmed to have a ratio of at least 1.00 for 28S/18S rRNA on the LabChips (Agilent Technologies, Inc.). RNAs used for aRNA synthesis and hybridization to chips were visually scanned for trends in the foreground median count in control sequences in the (x,y) coordinates. For the 4 λ-oligonucleotides spiked into each aRNA, the coefficient of variation (CV) in median foreground count was computed, and if the chip had a CV greater than 0.65 (n = 88), the sample was usually not used and redone. All chips were verified to have 51% of its features above median background (with the exception of samples 48 hrs, 44 hrs, 40 hrs, and 36 hrs on cycle 1, which were at 44–45%). This percentage (51%) of identified genes with expression above background is higher than that reported (38%) in [35] and close to the 60% reported in [36]. RT-PCR was done in parallel on all RNA samples, and if the relative quantification by RT-PCR with ΔΔC_□_ method did not agree quantitatively with the median foreground counts obtained on *wc-1*, *wc-2*, and *frq* from the Combimatrix chip hybridizations, the aRNAs and hybridizations to oligonucleotide arrays were redone. Two samples on cycle 1 and two samples on cycle 2 required redoing. Four samples of cycle 3 required redoing because of gradients on the foreground median counts on the arrays. Data are deposited in the *Neurospora crassa* functional genomics database at http://www.yale.edu/townsend/Links/ffdatabase/introduction.htm [34] under accession numbers 13 (cycle 1), 34 (cycle 2), and 36 (cycle 3).

#### Microarray Analysis

The median foreground (FG) counts were used on all 12K features. From each median foreground count on an oligonucleotide array a background subtraction was performed using the 5^th^ percentile of the following negative control oligonucleotide features: (1) plant; (2) bacterial; (3) phage not spiked into aRNA; (4) quality control oligonucleotides (QC); and (5) empty. Then the median foreground counts were normalized within arrays by multiplying each feature's median FG count on a particular chip x (median FG across all chips in the cycle) / (median FG count on the particular chip). A MIPS functional classification was assigned each feature on a chip [37]. Hierarchical clustering of genes was implemented using the methods proposed in [38] and implemented in Cluster 3.0 [39] available at http://bonsai.ims.u-tokyo.ac.jp/mdehoon/software/cluster. Options selected for analysis were log transformation, mean-centering and normalization followed by average linkage (*i.e.*, UPGMA) using Euclidean distance. Trees were displayed with Java TreeView 1.0.12 [40] available at http://treeview.sourceforge.net.

#### Searching for WCC and QA-1F binding sites in silico

Putative WCC GATX-binding sites or *Light-Response Elements (LREs)* were identified with the program pattern (Accelrys, Inc.) operating on the 1000 nt upstream of each identified gene [32] from file neurospora_crassa_#10BD2C.fasta from the Broad Institute Web site. The offset used was 1 and overhang 0. A mismatch of 2 was allowed. Patterns searched for were:

GATG{5,20}GATG{5,20}
GATC{5,20}GATC{5,20}
GATA{5,20}GATA{5,20}
GATT{5,20}GATT{5,20}.

Putative QA-1F-binding sites were identified with the program pattern (Accelrys, Inc.) operating on the 1000 nt upstream of each identified gene [32] from file neurospora_crassa_#10BD2C.fasta from the Broad Institute Web site. The offset used was 1 and overhang 0. Patterns [41] searched for were:

GGATAA{4}TTATCC, GGRTAA{4}TTATCC, GGGTAA{4}TTATCC, GGATAA{4}TTATCC, GGGTAA{4}TTAAGC, GGTTAT{4}TCATCC, GGATGA{4}TTAACC, GGCTAA{4}TTAACA, GGGTAA{4}TTTTCC, GGCAAA{4}TCATCC, GGATAA{4}TAACCC, GGGGAA{4}TTATAG, GGATGA{4}TTCTCC, GGCGAA{4}TTACCC, CGTTAA{4}TTATTC, and GGCTCA{4}TCATCA.

## Results

### Genetic networks for the biological clock guide the MINEing for clock-controlled genes

The genetic network models in Figs. 2 and 4 [7], [43] make three predictions about each gene in the genome under clock-control: a *clock-controlled gene* should: (1) maintain an endogenous circadian rhythm when the organism is grown in the dark; (2) be light-responsive when the organism is moved from Dark to Light (D/L); (3) change its expression when the level of the transcription factor WCC is dialed down (see Materials and Methods). The Computing Life paradigm is used below to discover these *clock-controlled genes*.

### Maximally Informative Next Experiment (MINE)

The objective of the MINE approach is to develop a quantitative criterion (or criteria) for the amount of additional information that can be gained about the genetic network from the “next” experiment to be performed; and then to maximize this “measure of additional information”, denoted by V(U), with respect to the choice of the design or “control” parameters of the next experiment, denoted by the control vector U. Control vector U comprises all those parameters which are known to, and are to some extent controllable by, the experimenter and which completely characterize measurements to be performed and the external conditions and perturbations applied to the biological system during the experiment. Two critical inputs for the MINE calculation are the underlying network kinetic rate equation model of the genetic network and any available “prior” or “old” experimental data. In a recently developed ensemble simulation approach [7], [11], these two inputs are combined both to constrain the unknown kinetics model parameters and to predict the likely information content V(U) for the next experiment, given U. Technical details and underlying conceptual ideas of the MINE approach are described in the Materials and Methods. Here we have used one of the MINE criteria in the Materials and Methods, V(U) = det(E(U)), Eq. (16), to guide the design of new experiments on the biology of the clock. This criterion is *the determinant of the ensemble correlation matrix E(U) between predictions*. The predictions here are of the log concentrations of *wc-1*, *wc-2*, and *frq* mRNAs over time in the next experiment.

When the predictions of two models in the fitted ensemble (the collection of all models consistent with available data) are highly correlated, the models will be difficult to distinguish by the next experiment U; when the predictions of two models in the ensemble are less correlated, they will be more easily distinguished in the next experiment U. A higher value of the MINE criterion V(U) recommends the experiment U for which predictions between any two randomly selected models in the fitted ensemble are more uncorrelated and hence more distinguishable. Each MINE calculation is done within the constraint of a fixed budget (*i.e.*, 13 microarray chips per experimental cycle or equivalently, 13 time points to be sampled). The budget and hence the number of time points determine the dimension of the correlation matrix E(U).

Two possible hypotheses have been developed for the clock mechanism in Figs. 2 and 4, from [7] and [33]. An older and slightly more realistic genetic network [33], [43] in Fig. 4 was used to guide the MINEing because the simpler genetic network in Fig. 2 was developed while the MINE experiments were underway. The older network [33] allows a light and dark form of WCC [4]. At the conclusion of the Computing Life enactment, these two different networks are tested against each other.

### Cycle 1 - Which genes are circadian?

The first series of microarray experiments were designed to determine how many genes are under clock-control. If such genes were outputs of the clock mechanism in Fig. 4, then they should be able to maintain an endogenous rhythm of ∼22 hrs (hrs) in the dark. The first experiment involves growing the organism in the dark for 48 hrs to observe the endogenous rhythm. The initial MINE design question concerns how often should we sample and when should we start sampling. The spacing between observations is denoted by t_S_, and the delay till the first observation by t_L_. The maximum in spacing (t_S_) is limited by the time over which circadian rhythms are maintained in liquid culture and the cost constraint of 13 microarray chips (see Materials and Methods). A MINE calculation using published data [3], [23]–[25] results in Fig. 5, based on the genetic network in Fig. 4.

The “best” experiment, with maximum det(E(U)) – in the upper back corner of Fig. 5 – is to start sampling immediately and to use the maximum spacing of 4 hrs between observations. This was the microarray experiment, performed (see Materials and Methods) with the results shown in Fig. 6. These experimental results would suggest that as many as 43% of the genes could be clock-controlled. A more detailed statistical analysis below reduces this percentage to 25%. There are 2436 (22%) circadian genes with *light response elements* (LREs) upstream [4] out of 11,000 genes, which is still considerably higher than 2–10% of circadian genes reported for *Drosophila* [44], [45] and *Arabidopsis* [46]–[48], and 10% higher than that reported in [8] for *Neurospora*. Our percentage, however, is not out of line with estimates of 36% based on *in vivo* enhancer traps in *Arabidopsis* [49].

In addition to the *11,000 N. crassa* genes on each chip (including 43 genes used as positive controls), the chips carried 633 negative control oligonucleotide sequences including those derived from plant, bacterial, phage, and *N. crassa rDNA* sequences. The empirical false positive and false negative rates are reported in Table 1 for each microarray experiment. For the first microarray experiment (cycle 1), the empirical false positive rate is 18% with the nominal significance level of the periodicity test used. Of these 4721 circadian genes, 2436 of them have a LRE upstream [4]. With a multiple test correction suggested for microarray analysis by Storey [50] and implemented as in Benjamini and Hochberg [51], the number of circadian genes with upstream LREs drops to 1460. In this multiple test correction the ranked list of genes sorted by their P-values is simply trimmed from the high end near the nominal significance level (of P-values) to control the False Discovery Rate (FDR) as described in [52]. The target FDR is set to the nominal significance levels in Table 1. If we subtract the 18% of false positives, then 43−18 = 25% of the genome would appear to be under clock-control. The estimated percentage of circadian genes (25% corrected for false positives) is close to the uncorrected percentage of circadian genes with LRE(s) upstream (22%). Requiring the presence of an upstream LRE appears to be a good filter for circadian genes.

**Table 1:** Observed fraction of false positives and false negatives among 633 negative controls on each microarray chip (see Materials and Methods) and among 43 distinct genes as positive controls using reported clock-associated genes [93].

Microarray Experiment	Nominal significance level (α)	Observed fraction of false positives	Observed fraction of false negatives	estimated power
**Circadian cycle (cycle 1)** (in the dark)	0.05	0.18	0.37	0.63
**Light-response (cycle 2)** (D/L)	0.20*	0.17	0.47	0.53
**WCC response (cycle 3)** (turn WCC off)	0.20*	0.22	0.44	0.56

The estimated power is 1 – fraction of false negatives observed. The fraction of false positives observed can be compared with the nominal significance level used to identify genes that are: (1) circadian; (2) light-responsive; (3) WCC-responsive.

* The nominal significance level was adjusted using the positive and negative controls to insure that the estimated power was high.

**Figure 7:**
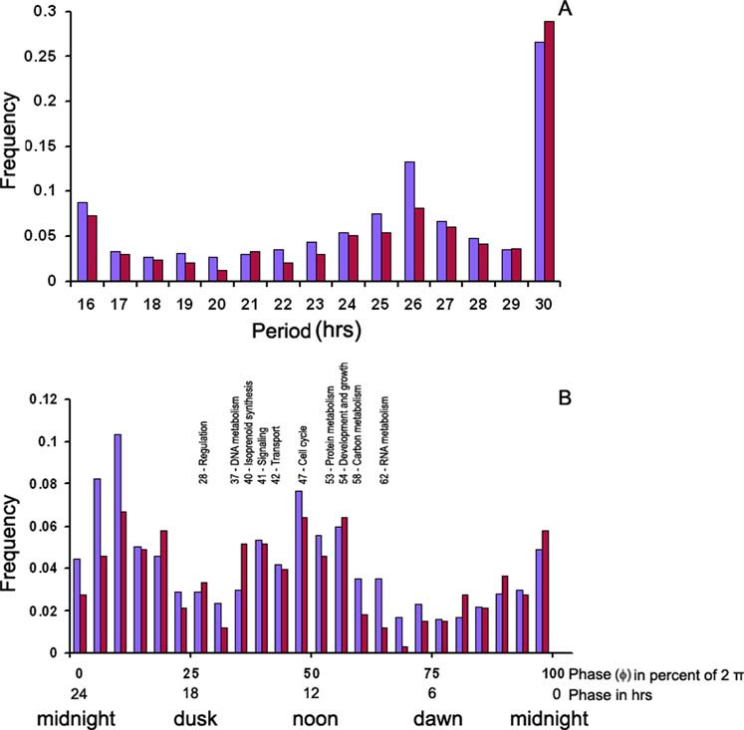
A. Blue bars show the frequency (count/2436) of 2436 genes in Fig. 6 by their period of oscillation in hrs. The mean period of oscillation is 24.9 hrs+/−0.09 hrs. B. Blue bars show the frequency (count/2436) of the 2436 genes displayed in Fig. 6 by their phase φ. A phase φ of 100% corresponds to a phase of 2 π radians. The phase is also reported in hours on a separate scale. For comparison, red bars show the distribution of period and phase for *clock-controlled genes* in Fig. 15 as well. In Panel B the mean phase of *clock controlled genes* by functional category from Fig. 15 [37] in cycle 1 is reported in the inset. Categories are defined in the legend of Fig. 15. While suggestive, an F_9,121_ of 1.81 from a one-way ANOVA between MIPS functional categories is not quite significant (P = 0.07).

Circadian oscillations are seen in Fig. 6 along rows by the alternating pattern of red (high expression) and green (low expression) for different genes, albeit with different phases. There also appear to be two clusters of known clock-associated genes with similar transcriptional profiles at the top and half way up Fig. 6 with different phases. The distribution of periods of oscillation is found in Fig. 7A with a mean of 24.92+/−0.09 hrs, implying the oscillations are circadian as predicted. This compares well with the average period between conidiating bands of 21.64+/−0.05 hrs obtained from 135 race tube assays (see Materials and Methods).

The phase of the *clock-controlled genes* is also interesting in Fig. 7B. Dusk (L/D transition) is taken as the zero time. The phase φ of 100% corresponds to being 360 degrees out of phase with genes at midnight. There are morning and evening genes in Fig. 7B. The *frq* mRNA has a phase of 48%+/−16% as a typical morning gene, and the *wc-1* mRNA has a phase of 69+/−12% as a typical dawn gene, being a blue-light receptor [4]. RNA metabolism genes tend to be dawn genes as well with a mean of 62+/−5% (Fig. 7B), while regulators tend to be dusk genes with a mean of 28+/−9%. Cell cycle genes *among clock-controlled genes* (in red in Fig. 7B) tend to be morning genes with a mean of 47+/−8%, as is the cell cycle checkpoint kinase *prd-4* [53]. This is consistent with light triggering conidiation in Fig. 1. The phase of genes may provide some clues as to how the clock allows the organism to adapt to its environment (see Discussion).

A naïve expectation might be that while the phase would vary between different *ccg*s, as in Fig. 7B, the gene periods in Fig. 7A would be expected to be the same. Several possible causes for this variation in period of *clock-controlled genes* in Fig. 7A present themselves. There is noise in mRNA profiling measurements on which the period estimates are based; there is also intrinsic noise in mRNA levels from cell to cell [54]; and we are observing the system over a short time interval covering only 2 periods of oscillation. As illustrated in [7], it can take longer than 2 periods before the limit cycle is established and, during the transient prior to that, neither period nor phase of oscillation are well-defined. The finite observation time also limits the accuracy of the measured period by way of an uncertainty principle [55]: the shorter the observation time, the greater the uncertainty in frequency and period.

**Figure 8:**
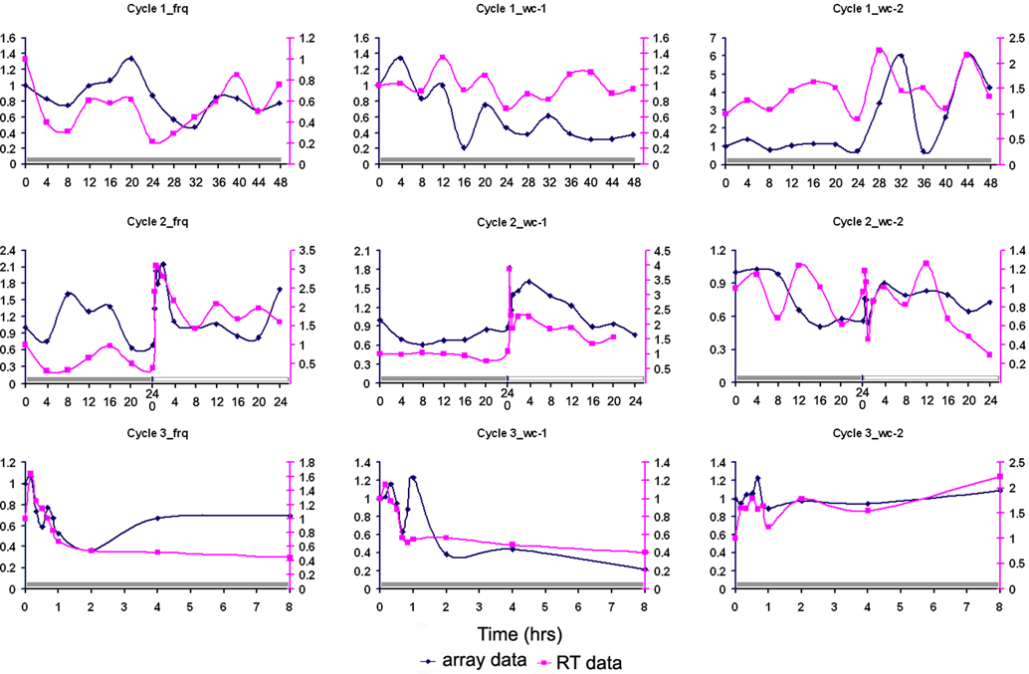
RT-PCR results for cycles 1–3 validate results of oligonucleotide arrays for *wc-1*, *wc-2*, and *frq* mRNA levels in cycles 1–3 of the Computing Life paradigm. The scale on the left is for fold expression change for oligonucleotide array measurements, and the scale on the right is fold expression change for RT-PCR results. rRNA was used as a standard in the RT-PCR experiments. Time on the x-axis is in hrs. Grey bars indicate lights off, and a white bar, lights on.

Cycle 1 microarray results were validated by RT-PCR (see next section) on twelve genes including *wc-1*, *wc-2*, and *frq* relative to an rRNA standard with excellent quantitative agreement (Fig. 8). The surprise was seeing oscillations in the *wc-2* mRNA with a period of 22.17 hrs+/−1.66 hrs, which have not been reported before (see validation in the next section). The presence of a LRE upstream of the *wc-2* gene would suggest adding additional feedback loops to *wc-1* and *wc-2* in Figs. 2 and 4 to make them autoregulatory. Evidence for an autoregulatory loop for *wc-1* has recently been provided [56]. Oscillations in *wc-1* mRNA are weak if plotted on the same scale as *frq* mRNA levels, as expected [24], [7]. The periods for *frq*, *wc-1*, and *wc-2* mRNA oscillations of 21.40+/−1.69 hrs, 23.5+/−2.47, and 22.17+/−1.66, respectively, in Fig. 8 agree well with the period of banding in race tubes above, namely 21.64+/−0.05 hrs [31], [33].

### RT-PCR confirms microarray results in cycle 1

The cycle 1 microarray results were validated by RT-PCR (see Materials and Methods) on twelve genes including *wc-1*, *wc-2*, and *frq* relative to an rRNA standard with excellent quantitative agreement (Fig. 8). This period of oscillation in *wc-2* mRNA (22.17 hrs+/−1.66 hrs) in Fig. 8 is not significantly different from that of the *frq* mRNA (21.40 hrs+/−1.69 hrs). The cycle 1 experiment was repeated in its entirety as well as the measurement of *wc-2* mRNA levels by RT-PCR with almost the same results (results not shown).

#### RNA metabolism genes

As a confirmation of microarray results on RNA metabolism genes, an entire replicate of the cycle 1 experiment was conducted (results not shown and same replicate used for validating *wc-2* microarray results), and the levels of *LHP1*, *PAB1*, and *ROK1* homologs' (denoted *lhp-1*, *pab-1*, and *rok-1* in *N. crassa*) mRNAs were measured every 4 hrs over a 48 hr window in the dark by RT-PCR (See Materials and Methods) in two replicates of cycle 1 (including the original cycle 1 experiment in the dark. A combined estimate of the amplitudes based on 26 time points was tested with an F-test (as described in the legend of Fig. 6) and found to be F_1,24_ = 5.55, P = 0.04 for the *lhp-1*, F_1,24_ = 3.60, P = 0.07 for *pab-1*, and F_1,24_ = 4.96, P = 0.04 for *rok-1*. All three genes had an estimated period of 17 hrs.

#### Regulators

An entire replicate of the cycle 1 experiment was conducted (results not shown and same replicate used for validating *wc-2* microarray results), and the levels of *kal-1* and *rpn-4* mRNAs were measured every 4 hrs over a 48 hr window in the dark by RT-PCR (see Materials and Methods) in two replicates of cycle 1 (including the original cycle 1 experiment in the dark). A combined estimate of the amplitudes based on 26 time points was tested with an F-test (as described in the legend of Fig. 6) and found to be significant (F_1,24_ = 4.95, P = 0.04 with a period of 23 hrs for *kal-1* and F_1,24_ = 6.65, P = 0.02 with a period of 30 hrs for *rpn-4*).

#### Signaling

The gene *rrg-1* has a role in the osmotic response signaling pathway [57]–[58]. An entire replicate of the cycle 1 experiment was conducted (results not shown and same replicate used for validating *wc-2* microarray results), and the levels of *rrg-1* mRNA were measured every 4 hrs over a 48 h window by RT-PCR (see Materials and Methods) in two replicates of cycle 1 (including the original cycle 1 experiment in the dark). The combined estimate of amplitude based on 26 time points was tested with an F-test (as described in the legend of Fig. 6) and found to be significant (F_1,24_ = 6.82, P = 0.02) with a period of 17 hrs.

### Cycle 2 - Which genes are light-responsive?

Each of these 2436 circadian genes from cycle 1 could be under the control of: (1) WCC; (2) a different oscillator [8]; or (3) multiple oscillators [59]; or be false positives. The chance of the latter is only 18% (see Table 1). As shown in Fig. 1, an important element to the clock is light-entrainment. As the organism is grown under different “artificial days”, that is, different periods of alternating light exposure, the organism speeds up or slows down its biological clock.

**Figure 9:**
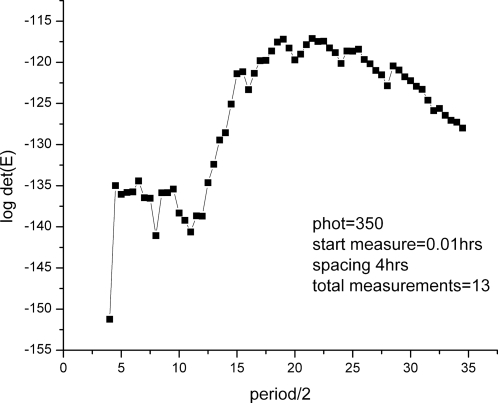
MINE calculation to determine what artificial day to use in cycle 2. Graph of the decadic log of the MINE criterion det(E) as a function of the half period of the artificial day in hrs. The calculation suggests trying a long artificial day with a half-period of daylight between 19 and 24 hrs of light. The inset gives: (1) the photon concentration of micromoles per second per meter squared (µM/s/m^2^); (2) the starting time (t_L_), which was selected to be close to zero but not zero to assist in the computation of the MINE criterion det(E)); (3) spacing (t_S_) in hrs between observations; and (4) the total number of time-points, at which mRNA levels were measured (the number of arrays used).

If these genes were under WCC-control, then they should also be light-responsive according to the genetic network hypotheses in Figs. 2 and 4. This poses the question of what artificial day period should be used for the experiment. A MINE calculation results in Fig. 9 using published results [3], [23]–[25] plus the data in Fig. 6 (cycle 1), *i.e.*, the MINE calculations are cumulative with respect to data already obtained.

**Figure 10:**
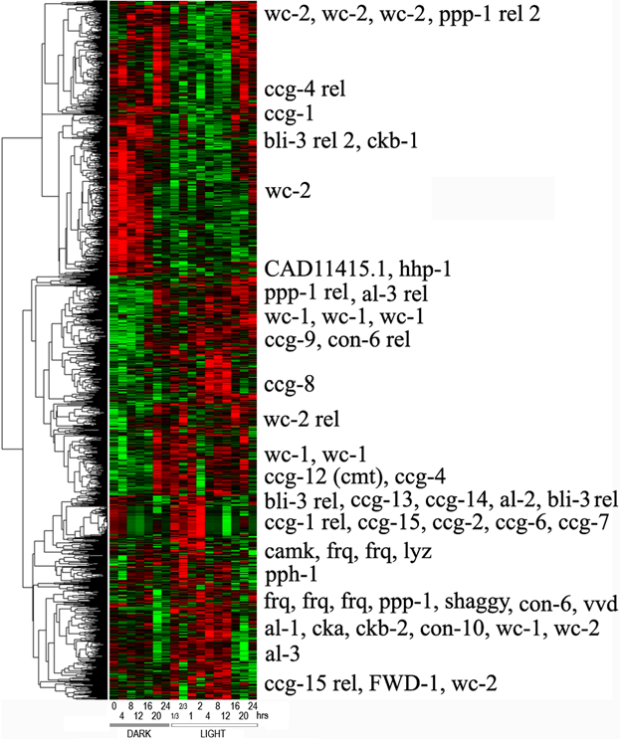
Transcriptional profile of approximately 1725 genes with LREs upstream at 0, 4, 8, …, 24, 24.3333, 24.6667, 25, 26, 28, …, 48 hrs (values staggered on x-axis) after shift from light to dark (L/D) followed by D/L transition 24 hrs later, after background subtraction, normalization within arrays relative to grand median of each chip, logging, and clustering with average linkage using Euclidean distance between mRNA profiles of different genes [38]. The bright green is −3, and the bright red is +3 is expression level on a decadic log scale. Data arose from 16 chips probed with a biotin labeled aRNA. Over 43 known clock-associated genes are overlayed in the right margin of this microarray experiment including varied known *ccg* genes. Genes in Fig. 2 are represented at least 5 times on each chip (explaining duplicate entries of *frq*, for example). The 1725 genes with upstream LREs were selected by a t-test comparing the mean of the first seven time points in the dark with the mean of time points 24.333, 24.6667, 25, 26, 28, and 32 hrs in the light with those having |t_11_|>1.363 (α = 0.20) displayed. The minimum observed t-value corresponded to a fold variation of 1.23 in the means before and after the light was turned back on. The mean observed t_11_ of the *frq* mRNA was 1.92. Grey bar denotes lights off; white bar denotes lights on.

The MINE results, shown in Fig. 9, suggest a long artificial day with a half-period of daylight of between 19 and 24 hrs. A second cycle of microarray experiments was therefore performed in which the light was turned back on after 24 hrs in a 48 hour observation period (see Materials and Methods). Results are shown in Fig. 10. Among these 3374 light-responsive genes (or 31% of *N. crassa* genes), 1725 (or 16% of the genes) responded to light in Fig. 10 and possessed LREs upstream. With the Benjamini and Hochberg [51] multiple-test correction, 1026 out of these 1725 genes with upstream LREs remain significant. In a similar experiment Lewis *et al.* [60] report detecting 22 light-responsive genes induced out of 1343 distinct genes arrayed as cDNAs, or 3%, and Ma *et al.* [61] report 34% of the unique genes in *Arabidopis thaliana* induced or repressed. Among the 31% of light-responsive genes detected here, up to 17% could be false positives, leaving 14% = 31%−17% as light-inducible. Among the 31% of light-responsive genes, 56% were induced (as opposed to repressed). The percentage of 0.56×14% = 8% is still higher than the 3% of Lewis *et al.* [60] (and less than the 31% of *A. thaliana*). The estimated percentage of light-responsive genes (14% corrected for false positives) is close to the uncorrected percentage of light-responsive genes with LRE(s) upstream (16%). Requiring an upstream LRE also appears to be a good filter for light-responsive genes.

**Figure 11:**
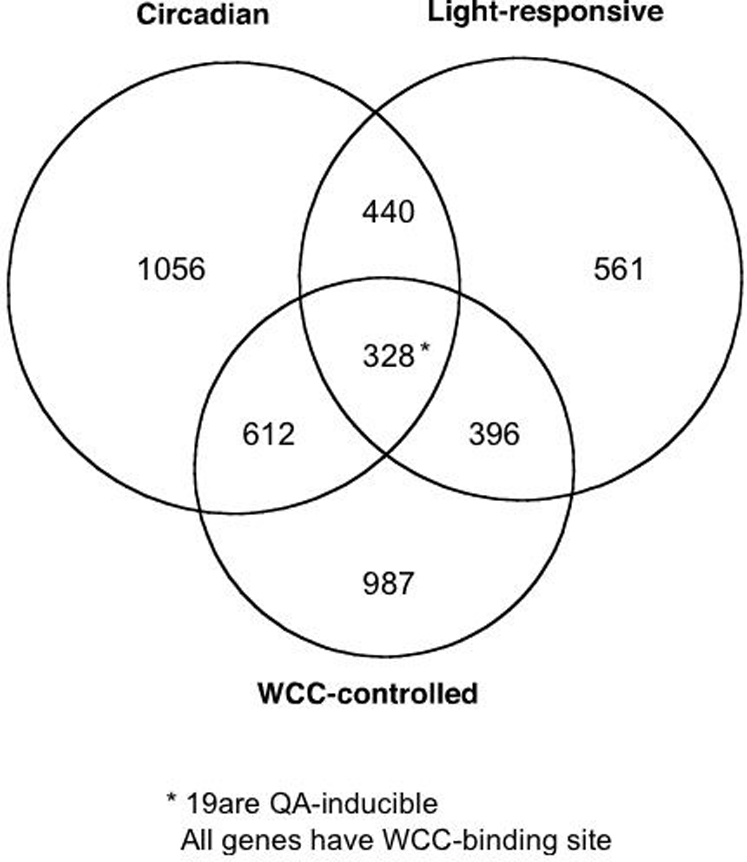
Classification of 4380 *N. crassa* genes with upstream LREs in a Venn Diagram by their response in each of the three microarray experiments: (1) cycle 1 (assay for circadian rhythm); (2) cycle 2 (assay for light response); and (3) cycle 3 (assay for response to changing levels of WCC). The diagram summarizes the microarray experiments in cycles 1–3 of the Computing Life Paradigm.

A total of 768 genes were both circadian and light-responsive (Fig. 11), and the chance that any one of these 768 genes is a false positive would be (0.18)×(0.17) = 0.03 (Table 1), since the experiments were done independently. These 768 genes then remain candidates for *ccg*s in Fig. 2 or 4. The response by genes to light falls into two clusters, one cluster being turned off (in the top part of Fig. 10) and one cluster being turned on (in the bottom part of Fig. 10). The positive response of some genes to light appears largely transient with a burst of expression after the Dark-to-Light (D/L) transition while other genes appear to have a sustained response after the D/L transition. Most of the known clock-associated genes fall within the bottom cluster of light-responsive genes with LREs upstream, as expected.

As a control, these results from cycle 2 were compared with a near replicate of this experiment reported in [60], using a different microarray technology and only a sample of the genes in *N. crassa*. With a power of 53% (Table 1) in our experiments, we would expect to see around 53% concordance with the experiments reported in [60]. In fact, we saw 64%+/−20% of the genes reported as light-responding in [60] in our cycle 2 experiments with good agreement to the 53% expectation.

### Cycle 3 - Which genes are under WCC-control?

Another prediction of the genetic networks in Fig. 2 or 4 is that if WCC were dialed down (*i.e.*, the mRNA level of *wc-1* is reduced by use of a QA-inducible promoter as described in Materials and Methods), then a gene under its direct or indirect control should experience a sudden change in its mRNA level. To test this with a gene knock-down experiment, it is necessary to ascertain first what gene should be perturbed to yield maximum information about the genetic network in Fig. 4. A MINE calculation was done using published results [3], [23]–[25] plus the data in Fig. 6 (cycle 1) and the data in Fig. 10 (cycle 2) as described in Materials and Methods.

**Figure 12:**
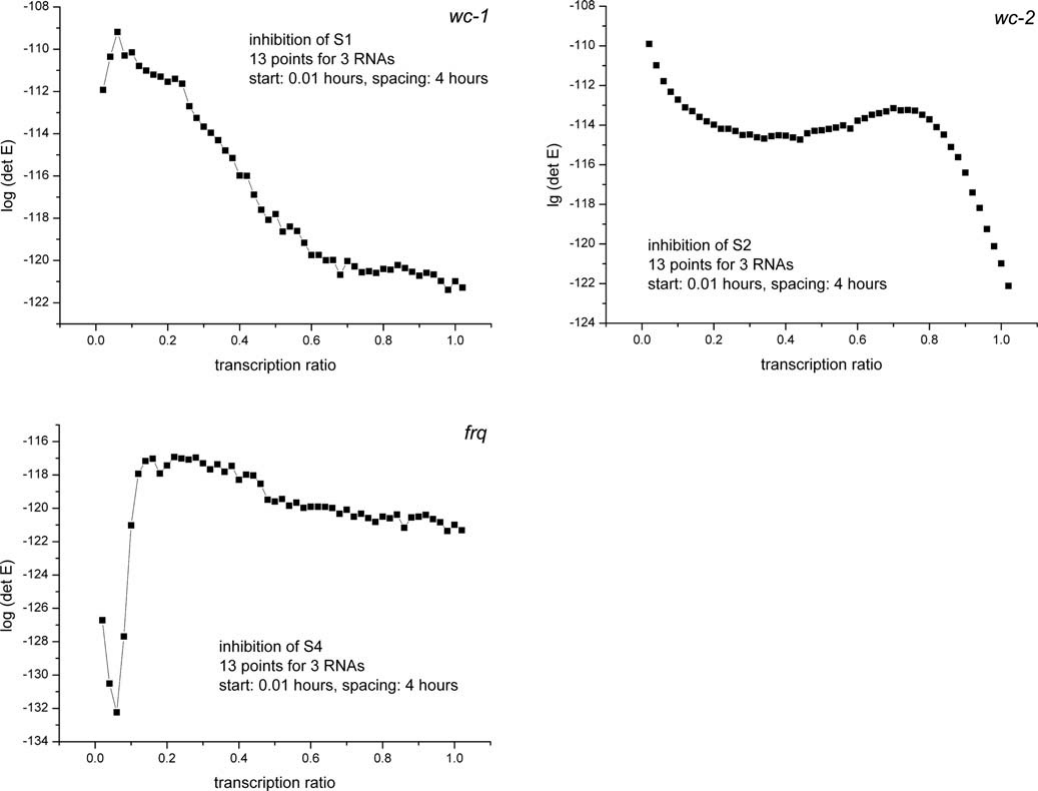
A 90% knock-down of the *wc-1* gene is the MINE experiment. The decadic log of the MINE criterion det(E) is displayed as a function of percent remaining activity of the three clock genes *wc-1*, *wc-2*, and *frq*. The matrix E is the correlation matrix of the predictions, emphasizing independence of predicted data points f(.,u_i_). The predictions are for the mRNA levels of *wc-1*, *wc-2*, and *frq* over time.

**Figure 13:**
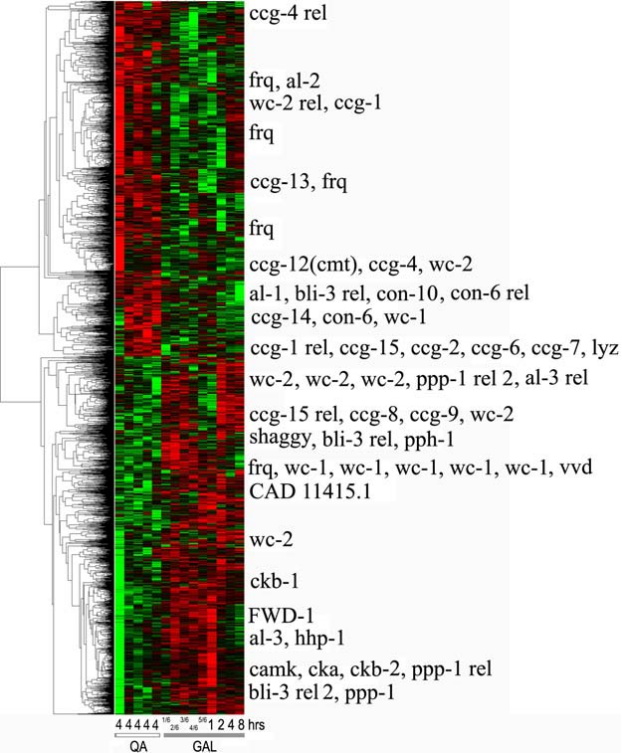
Transcriptional profile of approximately 2323 genes with upstream LREs at 0, 0.1667, 0.3333, 0.5000, 0.6667, 0.8333, 1, 2, 4, 8 hrs (time points appear on x-axis) after shift simultaneously from light to dark (L/D) and from quinic acid (0.3%) to galactose (2%) after background subtraction, normalization within arrays relative to grand median of each chip, logging, and clustering with average linkage using Euclidean distance between mRNA profiles of different genes [38]. There were 5 replicate zero time points (labeled on the x-axis with 4 hours of light before harvesting). The bright green is −3, and the bright red is +3 is expression level on a decadic log scale. Data below arose from 14 chips (including 5 replicate zero time points) probed with a biotin labeled aRNA. Over 43 known clock-associated genes are overlayed in the right margin of this microarray experiment including varied known *ccg* genes. Genes in Fig. 2 are represented at least 5 times on each chip. The 2323 genes with upstream LREs were selected by t-test comparing the mean of the first five time points on quinic acid with the mean of the last 9 time points on galactose with those having |t_12_|>1.356 (α⋅ = 0.20) displayed. The minimum observed t-value (1.357) corresponded to a fold variation of 0.70 in the means before and after *wc-1* was turned off. The mean observed t_11_ of the *wc-1* mRNA was −1.36. At the bottom of the heat plot grey bar denotes lights off; white bar denotes lights on.

The MINE calculation in Fig. 12, suggests that the most informative knock-down is to reduce *wc-1* to 10% of its original transcriptional activity. As detailed in Methods and Materials, the knock-down was engineered with a mutation in the native *wc-1* and with a quinic acid inducible copy of *wc-1^+^* introduced at another locus, producing a knock-down to 30% of its original activity; the results are shown in Fig. 13. A total of 4655 WCC-responsive genes were found to respond, but only 2323 of these genes had a LRE upstream as reported in Fig. 13. With the Benjamini and Hochberg [51] multiple-test correction, 1445 out of these 2323 genes with upstream LREs remain significant. The estimated percentage of WCC-responsive genes (20% corrected for false positives) is close to the uncorrected percentage of WCC-responsive genes with LRE(s) upstream (21%). Requiring an upstream LRE remains a good filter for WCC-responsive genes.

Most of the *frq* gene mRNAs were dialed down, as expected. The *frq* gene belongs to a cluster of genes being turned off in the top part of Fig. 13. Both *wc-1* and *wc-2* responded differently than *frq* and belong to the second larger cluster of genes being turned on (at least transiently) in the bottom part of Fig. 13. They have a fast transient response about 20–40 m after the QA/GAL (L/D) transition and then a drop off (see Fig. 8).

Also of interest are the 440 genes that are both circadian and light-responsive, but not under WCC control in Fig. 11. These 440 light-responsive and circadian genes which are not apparently under WCC-control, could be responding indirectly to genes under the control of WCC. They could also be false-negatives under the WCC-responsive assay (Table 1); they could be responding through a yet to be identified oscillator [8]; or they could be responding through multiple oscillators [59]. The chance that any one of them is a false positive is 0.03 = (0.18)×(0.17). The genes *cpc-1*, NCU05429, and *ccg-16* (WO6H02), have been previously identified as candidates for being under the control of another oscillator [8]. All three were found to be circadian here (confirming the results in [8]), and *cpc-1* and *ccg-16* were found to be light-responsive as well. All of them were found not to be WCC-responsive here, although in the case of *ccg-16* this appears to be a false negative [59].

Lewis *et al.* [60] conducted a related experiment that over-expressed *wc-1*. Only 18% of the induced genes corresponded to ones that we detected. A similar result was seen with overexpression of CLOCK in Drosophila [36]. There could be a variety of explanations, but it is not unexpected in a signaling system that over-expression leads to a different outcome than a knock-down, particularly when there are other coupled interacting pathways to those in Fig. 2 or 4. See, for example, the platelet-derived growth factor β receptor (PDGFRβ) signaling system [63] or the sonic Hedgehog (*shh*) signaling system in neurogenesis [64], where high and low levels of *shh* have different neurogenetic outcomes. The response of the clock to *wc-1* over-expression is apparently not the same as lowering *wc-1* expression. An additional MINE calculation analogous to Fig. 12 (results not shown) also suggested that that an over-expression experiment would not be as informative as a knock-down.

The Computing Life paradigm has led us to the discovery of 328 *clock-controlled genes* supported by all three series of microarray experiments and having an upstream LRE. Among these 328 genes, the chance of a false positive is (0.18)×(0.17)×(0.22) = 0.0067 (Table 1), the three microarray experiments having been done independently. A total of 104 of these 328 genes survive the multiple test correction in all three cycles [51]. These genes satisfy the three predictions of the genetic network and constitute *clock-controlled genes* (Fig. 11). Of these 328 *clock-controlled genes*, 314 of them are distinct on the arrays (some genes are represented multiple times; see Materials and Methods).

### Direct test of the auto-feedback loops activating wc-1 and wc-2

**Figure 14:**
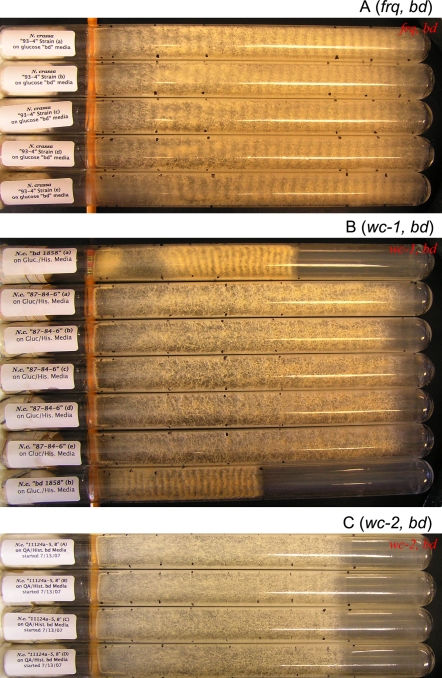
Light entrainment response under short artificial day by *frq* and *wc-1* mutations provides evidence for autofeedback loops on *wc-1* and *wc-2* in Fig. 2. (A) Light entrainment response of a *frq*, *bd* mutation (FGSC 93-4) during artificial days with 3 hrs of light followed by 3 hrs of dark in race tubes. (B) Light entrainment response of a *wc-1*, *bd* mutation (87-84-6) during artificial days with 3 hrs of light followed by 3 hrs of dark in race tubes. (C) Light entrainment response of a *wc-2^KO^*, *bd* mutation [65] during artificial days with 3 hrs of light followed by 3 hrs of dark in race tubes. A *bd* mutation was cultured in race tubes as a control. See Fig. 1 as well for “wild type”, namely the *bd* mutation.

All three cycles of microarray experiments support the presence of auto-feedback loops for WCC activating *wc-1* and *wc-2*. In cycle 1 there was evidence that *wc-1* and *wc-2* mRNAs were circadian in Fig. 8. In cycle 2 there was a fast light-response by *wc-1* and *wc-2* (of less than an hour) in Fig. 8. In cycle 3 both *wc-1* and *wc-2* were WCC-responsive in Fig. 8 and have upstream LRE elements. An experiment with a short 6 hr artificial day is predicted by a MINE calculation in Fig. 9 to be highly informative about the genetic network in Fig. 4. A prediction of the genetic network in Fig. 4 is that the auto-feedback loops added should permit entrainment to a short artificial day of 6 hrs duration (as in Fig. 1) independent of FRQ. To test this hypothesis, a strain (93-4) with a *frq* mutation was subjected to a short artificial day as seen in Fig. 14A. As can be seen, the rapid conidiation pattern with *frq* in Fig. 14A is indistinguishable from a *bd* mutant in Fig. 1. To rule out that the conidiation response to a short artificial day is under the control of an independent light-response pathway, a mutant in *bd*, *his-3*, *wc-1* (87-84-6) was generated by a cross, *bd his-3* (87-84)×*wc-1* (FGSC 3914). As can be seen (Fig. 14B), the *wc-1* mutation almost entirely removed banding under the artificial day of 6 hrs. To confirm this finding, the *bd*, *his-3*, *wc-1* (87-84-6) strain was transformed with a plasmid containing a QA-inducible *wc-1^+^* as described ([7] and Materials and Methods) and was found to band weakly when *wc-1^+^* was induced and not to band when *wc-1^+^* was not induced (results not shown). This establishes that banding under a short artificial day is under the direct clock control of *wc-1*. In a similar entrainment experiment a double mutant *wc-2^KO^*, *bd* from the cross *wc-2^KO^* (FGSC 11124, 65)×*bd* (FGSC 1858) was subjected to a 6 hr artificial day. As can be seen in Fig. 14C, *wc-2* also nearly removed all banding. As a final confirmation of these experiments, the *frq* gene was over-expressed on .001 M QA as well as turned off on glucose in a strain with a QA-inducible copy of the *frq* gene (see Materials and Methods), and these two conditions had no effect on the rapid banding (results not shown). These results serve to confirm that the rapid light response to a 6 hr day is due to the auto-feedback loops to *wc-1* (and *wc-2*) and not due to the FRQ oscillator.

### The clock mechanism of three genes wc-1, wc-2, and frq is pleiotropic in its effects on metabolism

**Figure 15:**
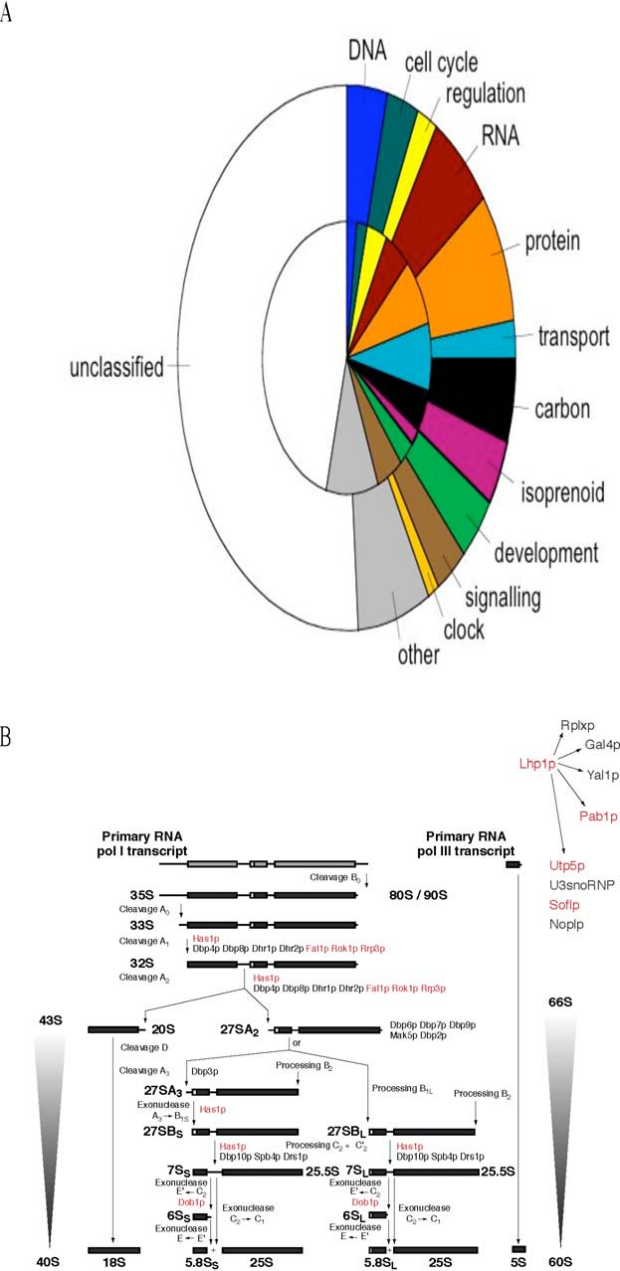
The clock of *N. crassa* has 295 distinct *clock-controlled genes* of diverse function as outputs. A. 328 Genes (with putative WCC binding sites) which are circadian, light-responsive, and WCC-responsive are classified by function (MIPS, [37]) in the outer wheel. 19 QA-inducible genes are not included in the outer wheel. With the exception of the unclassified category, the standard errors on the percentages of the outer wheel are 0.01–0.02. Gene products of the *N. crassa* proteome are classified by function as well (MIPS, [37]) in the inner wheel. The numbers below are a MIPS coding of functional categories. The definition of the categories [37] is DNA, 1.03, 10.01; cell cycle, 10.03, 10; transcriptional control & regulation, 1.02.04, 11.02.03.04, 16.0; RNA, 11.02, 11.04, 16.03.03; protein, 12.01,12.07, 14.04, 14.13, 14.07, 1.20; transport, 20.09, 20.03, 20.01; carbon and energy metabolism, 1.05, 2.01; isoprenoid, 1.06; development and growth, 40.01, 40.02, 41.01, 43.01; signaling, 30.01, 32.01, 32.05, 34.11; clock, *wc-1*, *wc-2*, *frq*; other, 16.19, 1.01, 1.02, 1.07,1.20, 2.04, 2.13, 2.19, 2.45,18.02, 34,70.01, 70.02, 70.04, 70.10; unclassified, 99 or no number. B. pre-rRNA processing. Proteins involved contain a ‘p’, and proteins in red are encoded by *clock-controlled genes*. An arrow from Lhp1p indicates that the encoding mRNA is a target of Lhp1p as well as the U3 snoRNA in *S. cerevisiae*. A, B, C, D are cleavage sites. Modified from Emery *et al.* [95].

These 314 *clock-controlled genes* identified are involved in a broad range of biological functions: DNA metabolism, replication, repair, cell cycle, RNA metabolism, transport, carbon and energy metabolism, isoprenoid biosynthesis (including carotenoids), development, and signaling (Fig. 15A). Periods and phases of all 295 (314–19; see below about 19) *ccg*s are similar in distribution to all circadian genes (Fig. 7) with upstream LREs.

The connection of the clock to development has been reported (*ccg-2*, *ccg-4*, *ccg-6*, and *con-6*). A recent connection to RNA metabolism has been through the *frequency RNA helicase* (*frh*) gene, whose product FRH co-immunopreciptates with FRQ [66]. The microarray experiments here identified *frh* and 16 additional genes in RNA metabolism under clock control (Fig. 15B). In addition to *frh*, 4 additional genes with products homologous to ATP-dependent RNA helicases in *S. cerevisiae*, namely *ROK1*, *HAS1*, *PRP16*, and *RRP3*, are among the 295 *clock-controlled genes*. At least three of these RNA helicases are involved in ribosomal RNA processing. While ribosome transcription is not under clock control (the ribosomal RNAs are not circadian in Cycle 1), almost all of the *ccg*s in RNA metabolism are involved in ribosome processing and assembly, *i.e.* ribosome biogenesis. These include *SEN2*, *SOF1*, *LHP1*, *RRP3*, *POP4*, *UTP5*, *RCL1*, *ABD1*, and *PAB1* homologs in *S. cerevisiae*. The yeast *PAB1* is a poly-A binding protein, and *LHP1* is another distinct RNA-binding protein involved in the maturation of tRNAs and snRNAs. *LHP1* has been implicated not only in the biogenesis of noncoding RNAs, but a recent ChIP/chip experiment in *S. cerevisiae* has demonstrated that it targets a number of mRNAs [67]. The RNase P (*POP4*) binds to the *RPR1* RNA, which is also a target of Lhp1p, and the *PAB1* mRNA is also apparently a target of Lhp1p in yeast as well [67]. Inada and Guthrie [67] also report enrichment of the gene encoding the snoRNA U3 among targets of Lhp1p. The product of *UTP5* is part of the processome containing the U3 snoRNA and involved in ribosome biogenesis. The clock's regulation of the ribosome appears to occur through its biogenesis rather than its transcription. This is a novel mechanism by which the clock can regulate *clock-controlled genes* post-transcriptionally.

Connections of the clock to DNA metabolism are recently reported in humans [68]. A human clock CLK2 protein physically associates with S-phase checkpoint components ATR, ATRIP, claspin, and the checkpoint kinase, Chk1. Also human CLK2-depleted cells accumulate DNA damage, engage in radio-insensitive DNA synthesis, and fail to recruit proteins, such as *RAD51*, functioning in human recombination pathways. Here several putative checkpoint-associated proteins (*e.g.*, NCU00560 and NCU04326) as well as 8 genes involved directly in purine/pyrimidine metabolism (NCU0 7590, 8359, 4323, 6262, 3194, 5542, and 4195) and repair (*uvs-6/NCU00901*) appear to be *clock-controlled genes*. The *uvs-6* gene is a homolog of *RAD50* in *S. cerevisiae* involved in double-stranded break repair. As predicted from the results on humans [68], the *RAD51* homolog (*mei-3*/NCU02741) was circadian and light-responsive in *N. crassa* in cycles 1 and 2, but not WCC-responsive (cycle 3).

The clock connection to the cell cycle has been only recently reported in Neurospora through *prd-4*, a homolog of Chk2 in yeast, a second checkpoint kinase [53]. In addition to *prd-4*, we have identified 2 other putative cell cycle checkpoint genes as *clock-controlled genes* (NCU00560 and NCU04326), homologs of *CDC4* and *CDC28* in *S. cerevisiae*. Up to 16% of rhythmic genes (cycle 1) may be involved in the cell cycle in some mouse tissues in contrast to the 3% in Fig. 15A identified by more stringent criteria (*i.e.*, positive in cycles 1–3) in *N. crassa* [69].

In that carbon metabolism showed up as significant and may have arisen due to the use of the QA-inducible switch in the last series of microarray experiments, one additional control was performed with wild type (OR74A – see Materials and Methods) in which many QA-inducible genes were identified with microarrays by a shift from 1.5% sucrose to 0.3% QA over a 0 to 8 hr window [11], [27]. Of the 314 distinct *clock-controlled genes* identified, only 19 of them were QA-inducible (with most of them being unclassified in function). Only 2 of the QA-inducible *clock-controlled genes* were involved in carbon metabolism. Subtracting the 19 QA-inducible *ccg*s from the 314 distinct *ccg*s, 295 *clock-controlled genes* remained.

**Figure 16:**
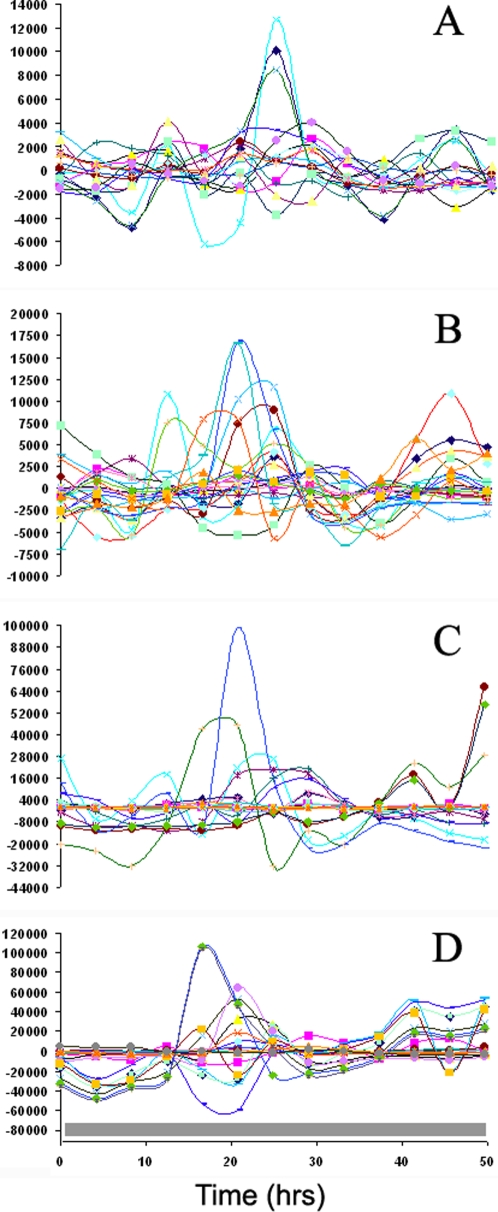
Transcriptional profiles of individual genes with upstream LRE elements in the dark (cycle 1) in the functional categories of: (A) regulation (MIPS functional classification categories 1.02.04, 11.02.03, and 16, [37]); (B) putative phosphatases/kinases (functional classification category 14.07.03); (C) signal transduction (categories 30.01, 32.01, 32.05, 34.11); (D) Development and growth (categories 40.01, 40.02, 41.01, 43.01). The mean mRNA level of each gene was subtracted from the 13 individual mRNA levels measured on each gene in Fig. 6. Data are from Fig. 6.

Approximately one-half of these 295 *clock-controlled genes* are of unknown function. The most prevalent known function among these genes are phosphatases and kinases. They make up almost half [11] of the 23 genes with products involved in protein synthesis (Fig. 16B), processing, and degradation, and at least three of the genes under DNA metabolism are known kinases/phosphatases as well (CK1, HHP1 homolog, and PP1). This plethora of phosphatases and kinases may reflect the role they play in modifying/linking the functions of *wc-1*, *wc-2*, and *frq* as regulators of (to) other pathways, as well as in the coupling of the clock to varied signaling pathways and the cell cycle. For example, the phosphatases PP1 and PP2a, dephosphorylate FRQ *in vitro*, thereby altering oscillator behavior [70], and the kinases CK1a and CKII mediate the phosphorylation of WCC [71]. After that, DNA metabolism, RNA metabolism, and carbon/energy metabolism represent equally important outputs of the clock. The clock outputs are representative of the frequency of these functions in the proteome [37] with two exceptions: a deficit of transport and unclassified genes that are *ccg*s.

While only one *ccg* in Fig. 15 has a product classified as a transcriptional activator (*PRO1* homolog) involved in fruiting body development (*kal-1*, NCU07392, [72]), four other *ccg* genes were classified as regulators. Their individual cyclical transcriptional profiles are given in Fig. 16A. One of these putative regulators is an inferred ornithine decarboxylase antizyme involved in sulfur and nitrogen regulation (NCU07155) [37]. This connection has also been reported in Arabidopsis [47], [73]. The remaining putative regulators identified under clock control in Fig. 2 or 4 were NCU00045, NCU01640 (*rpn-4*), and NCU06108.

Earlier work has suggested a link between signal transduction pathways for conidiation and the clock [74]. From microarray analysis here the clock is tied into a number of other signal transduction pathways as well, including stress (*ccg-9/NCU09559*), oxidative stress (NCU05169), light (*vivid/NCU03967*), mating (*ccg-4/NCU02500*, *NCU03378*, *NCU07335*), and osmo-sensing (*os-1*, *rrg-1*, *hpt-1*). The last output to the clock has only been reported recently (Fig. 16C). For example, *cut-1* involved in osmo-sensing has been reported to be under WCC control [75]. Jones *et al.* [57] have reported a role of *rrg-1* in osmo-sensing reminiscent of the *os* mutants. The genes *rrg-1* and *hpt-1* have an upstream LRE and were found to be circadian in cycle 1, but not light-responsive or WCC-responsive [58].

### Impact of standard that the period of a ccg is between 16 and 30 hrs

To provide insights on the impact of the standard [5] of requiring genes to have a period between 16 and 30 hrs to be declared *clock-controlled genes*, we tightened the standard to 17–29 hrs because of the up turn at the extremes of the distribution of periods in Fig. 7A. The result was declaring 2172 genes with upstream LREs as circadian as opposed to 2436 genes in Fig. 6. This reduces the number of *clock-controlled genes* identified by all three cycles of microarray experiments to 290. If the 14 duplicates of *frq*, *wc-1*, and *wc-2* as well as 18 remaining QA-inducible genes are removed, we are left with 258 *clock-controlled genes* with a range of periods between 17 hrs and 29 hrs. Genes affected in the text can be determined from their reported periods. Further trimming of the range of acceptable periods in Fig. 7A will gradually shrink the number of *clock-controlled genes* identified.

### Identifying an ensemble of genetic networks for the biological clock of N. crassa

The culmination of Computing Life is the identification of an ensemble of genetic networks describing how the clock functions from 3 cycles of microarray experiments initiated from published data [3], [23]–[25]. Results are summarized in Table 2. For 69% = (100×18/26) of the rate constants in common with Table 1 [7], standard errors were reduced by the addition of data from cycles 1–3. Measured lifetimes of the *wc-1* mRNA and FRQ protein remain concordant with estimated values in Table 2 with an order of magnitude increase in the amount of data (see Table 3). The long lifetime of the *wc-1* mRNA provided a critical test of the genetic networks [7], and the long lifetime of the *wc-1* mRNA of 7.4 hrs = D_7_^−1^ continues to be supported by microarray data here. Transcription rates of *frq*, A and 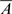, as well as the deactivation rate of WCC, P, were previously identified as critical parameters for maintaining oscillations through the negative feedback loop in Fig. 2 [7]. These constants are now more sharply defined in Table 2. Eleven of the 26 parameters identified in Table 1 of [7] are not significantly different from those in Table 2, although a majority of the rate constants are estimated more precisely. Precision of cycles 1–3 are assessed further in the next section and Table 3.

**Table 2:** Rate coefficients in the genetic network model (Fig. 4) of the biological clock (n = m = 4) based on data from cycles 1–3 predicting the clock's observed oscillations, light response, and *wc-1* perturbation (Fig. 17).

X	k	<X>	σ(X)
A	5	0.0313	0.00974
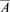	1	0.1108	0.00498
B	5	4.010E-4	1.020E-4
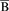	1	0.382	0.0412
S1	1	0.000420	0.0000048
S2	1	0.0220	0.00838
S3	1	5.47E-5	1.597E-4
S4	1	1.252	0.286
D1	1	6.607	1.399
D2	1	0.153	0.0247
D3	1	0.798	0.134
C1	2	1.047	0.220
L1	1	94.39	4.346
L2	1	0.3698	0.2207
L3	1	63.93	21.5
D4	1	0.00451	0.0118
D5	1	0.00890	0.00242
D6	1	0.205	0.00899
D7	1	0.135	0.0148
D8	1	0.0122	0.00304
C2	2	3.322	0.912
P	5	0.2233	0.2701
A_c_	5	0.1293	0.0826
B_c_	1	0.6091	0.1718
S_c_	1	2.572	2.757
L_c_	1	3.664	8.993
D_cr_	1	0.579	0.137
D_cp_	1	0.5536	0.1173
E1	2	0.003125	9.865E-4
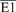	1	0.0965	0.0104
E2	2	2.614	2.607
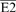	1	0.0128	0.0298
S5	1	8.924	0.696
D9	1	1.234E-4	3.259E-4
A_cL_	5	0.0524	0.0156
Q	5	4.812E-4	6.111E-4
D_10_	1	2.865E-4	9.257E-4
C_3_	2	5.5593	1.7937
Bc_L	1	0.00576	0.00633
Sc_L	1	0.07454	0.1344
E3	2	0.00974	0.00288
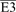	1	0.000542	0.00188
E4	2	1.335E-5	3.456E-5
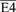	1	0.0121	0.00682

Ensemble mean <X> and ensemble standard deviation σ(X): = [<X^2^>−<X>^2^]^1/2^ for rate coefficients (*X*) in the *n* = *m* = *4* biological clock model of Fig. 4. For a *k* ^th^ order reaction (with *k* = *1*,*2*, or *5*), the rate coefficient is given in units of 1/(hour×cu*^k−1^*) where “cu” represents the arbitrary, but common model unit of concentration for all species, except for the *photon* species where 1 cu(photons) = 0.20 µmole(photons)/(s·m^2^), see also Materials and Methods. The estimated value of 1/〈*D*_6_〉≈5 hrs is consistent with the FRQ protein life-time of ≈4–7 hrs, estimated from the FRQ-decay data of [96].

**Table 3:** The quality of fit of the model usually improves in successive cycles through the Computing Life paradigm. Several measures of fit are reported.

Profiling Experiment	n	χ^2^_min_	σˆ^2^ $	GEL_50_ *	fold-change
**data from literature (cycle 0)**	333	1188	0.0714	-	-
**circadian cycle (cycle 1)** (in the dark)	553	2918	0.1055	2.82%	-
**light-response (cycle 2)** (D/L)	1927	3938	0.0409	1.97	1.89
**WCC-response (cycle 3)** (turn WCC off)	2165	5528	0.0511	1.97	2.48
**genetic network – ** **Fig. 2** ** (cycle 4)**	3007	4640	0.0309	-	-

The number of data points (n) used in fitting, χ^2^ goodness of fit measure [7], which is cumulative across cycles, estimates of the error variance σ^2^, Gene Expression Level 50 (GEL_50_)^*^ as a proxy for power [77], and the fold-change in expression level corresponding to the GEL_50_ are reported to allow comparison with future and existing models and microarray experiments. The genetic network fitted is shown in Fig. 4 (except cycle 4).

* The gene expression level 50 (GEL_50_) was the median value of the test statistic among genes with a significant F or t statistic for a circadian, light-, or WCC-response and with LREs upstream. This measure is an indicator of power [77] to detect one of these three responses, allowing comparisons with other microarray experiments. The fold-change in expression level (*e.g.*, D/L) corresponding to the reported GEL_50_ is reported in the last column when applicable.

$ The estimated error variance was computed from the number of observations (n), the preliminary estimate of σ_0_^2^ = 0.02 [7], and the χ^2^_min_ over the ensemble using the formula: 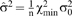.

% The square-root of this F_1,9_-statistic is reported using the fact that F_1,9_ = t_9_^2^ to allow a comparison with other t-statistics in the same column.

**Figure 17:**
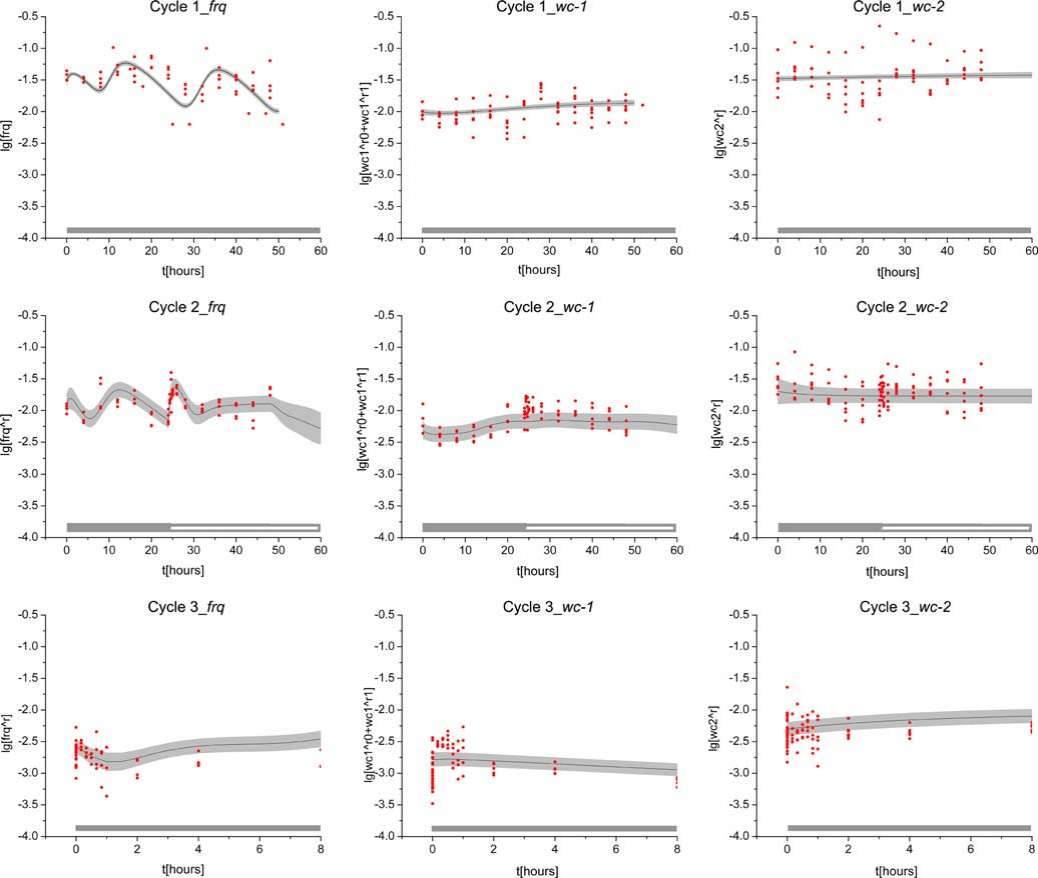
An ensemble of genetic networks predicts the mRNA levels of *wc-1*, *wc-2*, and *frq* for cycles 1–3. The decadic log (lg) of each gene's mRNA level is measured at least 5 times on an array for each time point. Some data points are from the literature [3], [23]–[25]. The curves represent the mean prediction of the ensemble of genetic networks in Fig. 4 +/− 2 ensemble standard errors about the ensemble mean. In Fig. 8 the averages of the 5–6 replicates of each mRNA level at each time points are displayed. Grey bars denote lights off; white bars denote lights on.

The behavior of the ensemble is displayed in Fig. 17. In cycle 1 the predicted oscillations of *frq* mRNAs are displayed with microarray measurements. The predicted oscillations in *wc-1* and *wc-2* mRNAs are much reduced relative to the *frq* mRNAs. In cycle 2 in the first 24 hrs the measurements and predictions track those in cycle 1; the correlation over all 87808 = 12544×7 microarray features between cycles 1 and 2 for the first 7 time points is 0.82. When the light is turned back on at 24 hrs into the cycle 2 experiment, the coordinated response of the ensemble and microarray data (particularly the *frq* mRNA) to light can be seen as the clock resets [62]. In cycle 3, the slow decline in the *wc-1* mRNAs is seen corresponding to a lifetime of 7.4 hrs = D_7_^−1^. An alternative ensemble in Fig. 2 from Yu *et al.* [7] was tested and performed about the same as the genetic networks in Fig. 4; the distribution of chi-squared statistics [7] for the ensemble fitted to Fig. 2 is largely overlapping with the distribution of chi-squared statistics for the networks in Fig. 4 (results not shown). By Occam's Razor the simpler network with fewer parameters in Fig. 2 is then preferred. The series of model-guided experiments now has identified and selected an ensemble of genetic networks describing the clock mechanism in Fig. 2.

### Comparison of the precision and power of microarray experiments in cycles 1–3 with other microarray experiments

A standardized way of assessing progress in the Computing Life paradigm as well for comparing the power of different microarray experiments here with others in the literature and in the future would be useful. Progress here is measured by the error per observation σ^2^ or *error variance*.

In linear and nonlinear models a standard approach to estimating the precision of an experiment is to estimate the error variance σ^2^ [76], as it appears in the likelihood for the genetic network [7]. Townsend [77] illustrates by simulation and data analysis that such a common variance component can be extracted from each of a variety of microarray experiments and used to compare different experiments. Under the multivariate Gaussian assumption leading to the likelihood in [7], a simple estimator for the error per observation σ^2^ can be constructed for successive cycles of the Computing Life paradigm:
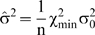
where n is the number of observations, χ^2^_min_ is the minimum chi-squared statistic over the ensemble [7], and σ_0_^2^ is a preliminary estimate of the error per observation in the multivariate Gaussian likelihood [7]. This preliminary estimate of σ_0_^2^ was allowed to vary across observations. In the preliminary data drawn from the literature [3], [23]–[25], σ_0_^2^ is 0.02 for the genetic network in Fig. 2 [7] and used on the RHS of expression above. Preliminary estimates of σ_0_^2^, 4σ_0_^2^, and 36σ_0_^2^ for published data [3], [23]–[25], microarray data in cycles 1–3, and for conidial banding data from Fig. 1, respectively, were used in calculating χ^2^_min_. These weightings were selected to give equal weight per time to different experiments in the ensemble fitting process.

In Table 3, the progress in reducing the error variance in successive cycles through the Computing Life paradigm is reported. The fourth cycle began with a switch to the genetic network in Fig. 2. In cycle 4 all experiments in different cycles were allowed to have their own initial conditions for initial species concentrations. An additional 842 data points of conidial banding data were collected under the regimen of a 48 hr artificial day (cycle 2). A downward trend in the estimated error variance across cycles is evident.

The final estimate of σ^2^ is 0.03, slightly larger than our initial guess based on published data from Northerns, Westerns, and race tubes [3], [23]–[25]. The advantage of having this estimated error variance is that it can be readily compared with other families of models, such as simpler linear models, used in microarray analysis [77] as well as to other experiments by other laboratories. The estimated error variance also allows diagnosis of whether or not further experiments will refine the model ensemble. Based on the downward trend in the estimated error variance further cycles would be predicted to be profitable.

In each cycle of the Computing Life paradigm we constructed a test statistic (F or t) for a response on a gene by gene basis and calculated the same for all genes with LRE elements. Imagine extracting a ranked list of these significant statistics in a particular cycle. Townsend [77] has shown that the median value of this significant test statistic in this list is a good proxy for power from simulations. This statistic is called the gene expression level 50 (GEL_50_). With each GEL_50_ statistic, there can be an associated fold-change in expression level that can often be substituted for the original statistic for ease of interpretation. The advantage of this GEL_50_ statistic is that it allows easy comparison across experiments reported in the literature and in the future. The GEL_50_ is reported for cycles 1–3 in Table 3. These values are in the range of at least 5 other microarray studies [77].

## Discussion

### What we know and do not know about clock-controlled genes

Model-guided microarray experiments through MINEing have revealed much of what we know and do not know about the biological clock in Fig. 11. At the center of the Venn Diagram there is a highly cross-validated set of 295 distinct *clock-controlled genes* behaving as the clock mechanisms in Fig. 2 or 4 would predict. To date, only sixteen *clock-controlled genes* have been discovered in *N. crassa* in over 40 years of clock biology [59]. This set of 295 genes is circadian, light-responsive, and under WCC-control and spans a broad array of functions (Fig. 15). It is quite remarkable that only three genes, *wc-1*, *wc-2*, and *frq*, could have such diverse and pleiotropic effects on the organism's transcriptome, and the full extent of the clock's role in the metabolic web has not been evidenced till now.

The series of model-guided array experiments also point to an intriguing set of 440 genes or 57% ( = 100×440/(328+440) in Fig. 11) of the genes that are circadian and light-responsive and not under direct WCC control. No more than half of these 440 genes can be explained by false negatives in the cycle 3 experiment assaying for WCC control according to Table 1. These genes could be under the indirect control of the clock mechanism. Some candidate regulators implicated by all three microarray experiments include five genes, NCU00045, NCU01640 (*rpn-4*), NCU06108, NCU07155, and NCU07392 (*kal-1*). One of these may be a regulator of nitrogen and sulfur metabolism (NCU07155), and another is a homolog to PRO1 (called *kal-1*), a homeo-box containing transcription factor with a typical *GAL4*-like DNA binding domain involved in fruiting body development [72]. Another possibility is that some of these mRNAs are modified post-transcriptionally by RNA-binding proteins which are *ccg*s, such as the *LHP1* and *PAB1* homologs, to control expression. In that RNA-binding proteins appear to have specific mRNA target populations [78], they may provide another mechanism for the combinatorial clock control of gene expression post-transcriptionally.

There are WCC-responsive genes that are circadian only (612), but not light-responsive as well as those that are light-responsive only (396), but not circadian in Fig. 11. The genes, which are circadian only and not light-responsive, could be explained by other regulators involved with the clock mechanism that suppress a light-response (or be under the control of an oscillator not connected to the light-response through WCC as suggested by Correa *et al.*, [8]). The possibility of an oscillator not connected to WCC is discussed in the next section below. Similarly, genes that are light-responsive only, but not circadian, might also be explained by regulators of the light-response that interact with WCC to repress a circadian response (or be part of a light-response pathway not connected to the known clock mechanism, but see below). One potential list of candidates as modulators of the circadian- and light-response are the 11 phosphatases and kinases that are *clock-controlled* genes in Fig 15. These two functions of being circadian and being light-responsive *can be separated* in the action of WCC through the outputs of the clock. Other kinds of posttranslational modifications of histones could be involved in chromatin-remodeling of upstream sequences to *clock-controlled genes* to determine gene activation through WCC [79].

Data on *wc-1* cis-regulation by Kaldi *et al.* [56] and on *frq* cis-regulation by Belden *et al.* [79] suggest a simpler hypothesis that invokes only one new regulator class. The *wc-1* gene has at least two kinds of promoters upstream responding to WCC. The CCAAT box promoter in front of *wc-1* is not light-responsive, while the TAATTA promoter in front of *wc-1* for WCC is light-responsive. The LREs determine light-responsiveness [4], while other genes, such as *clockswitch* (*csw-1*), regulate accessibility to promoters through chromatin-remodeling around the *Clock Box* or *C* box upstream of *frq* to determine the endogenous circadian rhythm. Froehlich *et al.* [80] have shown that the C box is necessary and sufficient for endogenous circadian expression of *frq*. An additional LRE more proximal to *frq* is light-responsive [4]. Genes (295 out of 328) that have both types of elements, C boxes and LREs, might be circadian and light-responsive. Genes (612) with a C box and no LRE might be circadian but not light-responsive. Light-responsive genes (396) could be explained by only having the LRE upstream. This alternative would also not require any new regulators beyond the enzyme *CSW-1* enabling chromatin- remodeling around the C box. In Table 4 there is a significant association of genes being light-responsive and not circadian vs. being circadian and not light-responsive and the number of upstream LREs. Unfortunately, there was no difference in the incidence of the known Clock box among the circadian/non-light-responsive (612) versus the 396 light-responsive/noncircadian genes. Further characterization of Clock boxes in front of other genes besides *frq* appears warranted.

**Table 4:** There is an association between whether or not a WCC-responsive gene is light-responsive and the number of its LREs upstream.

Gene Response		Number of LREs upstream				
		1	2	3	>3	N
**circadian/no light-response**	**O**	405	137	47	23	612
	**E**	390.94	139.65	56.36	31.33	
**light response/not circadian (cycle 2)**	**O**	228	91	46*	30*	396
	**E**	248.29	90.15	36.38	20.23	

WCC-responsive genes are classified as circadian and not light responsive or not circadian and light-responsive and by their number of putative upstream WCC-binding sequences (GATX sequences) or *LREs*. Observed (O) and Expected (E) counts of these genes under the hypothesis of no association are reported along with the chi-squared test of no association. See Fig. 11 for categories of genes being compared (N = 612 or 396).

* The chi-squared statistic is X^2^ = 12.8445 with df = 3 and critical value C = 11.34 for α = 0.01. The residuals (O–E)/sqrt(E) of the starred counts make a large contribution to the X^2^.

Fig. 11 would suggest considerable limits on what we know about the clock mechanism. There are 1056 genes that are circadian only and not WCC-responsive and 561 genes that are light-responsive only and not WCC-responsive, and no more than half of these can be explained as false negatives in cycle 3 (WCC response array experiment). These two sets of genes are not explainable directly by *WCC*. A similar observation was made on CLOCK in Drosophila [36]. This percentage of the unexplained has increased from the earlier array experiments carried out by Lewis *et al.* [60]. This would suggest that there are other genes involved in the biological clock mechanism and other genes involved in a light response.

From the model-guided array experiments there is a concluding suggestion on a new direction for MINEing for *clock-controlled genes*. There are 987 genes that apparently are not circadian or light-responsive, but under WCC control. The clock is well-known to respond to a variety of environmental cues or zeitgebers, such as temperature [81]. The fact that these 987 genes do not respond to light would suggest future cycles of discovery to examine how these 987 genes respond to temperature [82].

### The circadian response of genes with upstream LREs is stochastically independent of their light response conditional on the WCC response

The microarray experiments in cycles 1–3 allow us to infer a broad relationship of circadian, light, and WCC- responses by different genes in the genome from Fig. 11. We have established in Fig. 11 that the circadian and light-responses can be separated by the response of clock-controlled genes. It is natural then to ask how are these responses related in different genes? For example, we can ask if the circadian and light responses of different genes with LREs upstream are stochastically independent across all genes with an LRE element. A simple contingency table model in which the circadian and light- response of each of the 5702 genes with LRE elements are conditionally independent given the WCC response can be fitted as shown in Table 5 fairly well. The resulting goodness of fit chi-squared statistic X_2_^2^ is 7.20, which is barely a significant departure from the model at the 0.05 level. What this implies is that the circadian and light responses of genes with LREs upstream are almost entirely explained by whether or not they have a response to WCC. When the WCC response is off, then the circadian response of a gene with an upstream LRE is more probable (p_C_>q_C_ in Table 5). When the WCC response is on, then the light response of a gene with an upstream LRE is more probable (q_L_>p_L_ in Table 5). This would suggest that any gene, such as *csw-1* [79], acting on the light or circadian response of a gene with an upstream LRE would need to act through WCC. The only explanation for light and circadian responses of *ccg*s in Fig. 2 or 4 is WCC. If there were another oscillator acting on genes with upstream LREs, then its outputs would be constrained to behave in the same way as the known clock mechanism. That is, an additional oscillator would be coupled to the known clock mechanism in Fig. 2 through WCC and would have an independent light and circadian gene outputs conditional on a gene's response to WCC. If there were additional information, such as the number of upstream LREs (as in Table 4), then more could be said. This conditional independence result does not hold across all 11,000 genes, if the same model in Table 5 is fitted to all genes (X_2_^2^ = 771.70, P<0.001).

**Table 5:** The circadian response of each gene with an upstream LRE is conditionally independent of its light response.

Gene Response		Not WCC-responsive		WCC-responsive		
		NL	L	NL	L	
**not circadian**	**O**	1337	561	986	396	
	**E**	1338	560	957	425	
**circadian**	**O**	1056	440	612	314	
	**E**	1055	441	641	285	
						N = 5702

The independence is achieved by conditioning on the WCC-response. Genes are classified as “not circadian” or circadian, “not light-responsive” (NL) or “light-responsive” (L), or not WCC-responsive or WCC-responsive. Observed (O) and Expected (E) counts of these genes under the hypothesis of no association between the circadian and light responses conditional on the WCC-response are reported along with the chi-squared test of no association. See Fig. 11 for counts of genes by category of response. The no response category was computed as 5702−4365 (sum of counts in the remaining 7 categories). All duplicates of *wc-1*, *wc-2*, *frq*, and other genes were removed from the observed counts of genes (O) with upstream LREs in Fig. 11 to be classified into 8 categories below. The probability estimate of a gene being WCC-responsive is r = (2308/5702). The probability estimates of a gene being circadian or light-responsive when it is known not be WCC-responsive are p_C_ = (1496/3394) or p_L_ = (1001/3394), respectively. The probability estimates of a gene being circadian or light-responsive when it is known to be WCC-responsive are q_C_ = (926/2308) or q_L_ = (710/2308). These probability estimates can be used to compute the expected (E) counts below.

As an example 441 = p_C_×p_L_×(1−r)×5702.

* The chi-squared statistic is X^2^ = 7.20 with df = 2 and critical value C = 5.99 for α = 0.05.

### Clock as adaptation

The *clock-controlled genes* and their time of action in Fig. 7B provide a possible narrative on adaptation. This complex trait controls levels of asexual reproduction as shown in Fig. 1. The complex trait has a clear genetic basis in Fig. 2. Variation in the clock trait in natural populations has been demonstrated in *D. melanogaster* [83], *A. thaliana* [84], and *N. crassa* [85]. There may be the opportunity for natural selection to act on the clock network [86] through simple sequence repeats, for example, in WC-1 and FRQ [85]. It is clear that the clock controls fecundity in the asexual part of the life cycle in Fig. 1. In addition, longevity is in part under clock control. The *bd* mutation as an allele of *ras-1* [87] is a well-known longevity gene in *S. cerevisiae* [88]. In addition, the longevity assurance gene *LAG1* homolog [89] (designated NCU00008 in *N. crassa*) is under WCC-control from the cycle 3 experiments. There appears to be a strong connection between the clock and the fitness components of fecundity and longevity, as has been reported in other model systems [90].

The organism needs to protect its DNA from light. Light triggers the onset of conidiation at dawn, thereby placing the DNA into these environmentally robust packages, spores. Prior to cell cycle initiation in the morning to produce the spores, DNA synthesis must be reinitiated and completed before entry into cell-cycle checkpoints on the following day (Fig. 7B). The development of structures to produce these spores requires carbon and energy as well as activation of the developmental program in the morning (Fig. 7B) as well as some initial groundwork by regulators “as early immediate genes (IEGs)” in the evening. Implementation of the developmental program is timed through *clock-controlled genes* positioned in the Central Dogma played out in ribosome assembly, protein synthesis, modification, and degradation taking place in the morning. One of the features of spores that make them so resistant to environmental insults is the synthesis and incorporation of the isoprenoids as pigments. Apparently their biosynthesis is sufficiently important that the organism continues working on these protective factors in the afternoon. The clock mechanism can tune the phase of *clock-controlled genes* by adjusting the rate constants (particularly the degradation constants of CCGs) in the genetic network for *ccg*s in Fig. 2 as well as their level of expression. Setting the phase may set the level of WCC experienced by the *ccg* as shown in (Fig. 7B). This adaptationist interpretation of the role of different *clock-controlled genes* helps to explain why the clock orchestrates such a diverse set of players in the cell.

### Using the Maximally Informative Next Experiment (MINE) and its Consequences

While the MINE is the next experiment U* to give us the most new information about the network in Fig. 4, U* is defined within a set of possible experiments that are ultimately specified by the goal(s) of the experimenter. Within the constraint of a particular biological goal, such as finding *clock-controlled genes*, finding the MINE U* provides an avenue to obtain the most information about the genetic network in each successive cycle through the Computing Life paradigm. This still leaves the choice of a set of possible experiments, from which U* is drawn, in the hands of the experimenter. In the present case, the biological goal of identifying *clock-controlled genes* sets the stage on which MINE plays. Identifying *clock-controlled genes* leads us to ask the genetic network in Fig. 4 for their predicted behavior, thus establishing the set of experiments in cycles 1–3 to be considered. In this context MINE is a tool to achieve a particular biological goal. One could naively make the biological goal coincident with the unconstrained objective of learning the most about the genetic network, the criterion of MINE, but then the set of possible experiments U becomes very large and the optimization of V(U) computationally intractable. Adopting a particular biological goal, such as finding *ccgs*, puts structure on the design question and thereby enables the researcher to parameterize the optimization of the next experiment U.

There have been several consequences to the use of model-guided discovery by MINE through the choice of experiments. In detecting circadian rhythms in cycle 1 MINE involved a design with even spacing between observations of 4 hrs and with sampling starting immediately without delay (Fig. 5). This design in cycle 1 conforms to practice in previous experiments [5]. However, in detecting the light-response in cycle 2 and the WCC-response in cycle 3 there is a departure from conventional wisdom. In cycle 2 prior experiments on light-entrainment, researchers have avoided a very short 6 hr artificial day [25], and as a consequence have missed an opportunity to examine the autofeedback in the networks as in Fig. 14. In detecting a response to perturbations in the clock mechanism in cycle 3, conventional wisdom would have us focus on perturbing the oscillator gene *frq* [8]. In contrast the MINE calculations in Fig. 12 pointed to engineering mutations in *wc-1* to obtain more information about the network. Even when a mutation in the transcription factor WC-1 or CLOCK was pursued [36], [60], experimenters have elected to overexpress the transcription factor. The result has been a paucity of responding genes [36], [60]. The MINE calculations in Fig. 12 suggested that a knockdown would be more informative about the genetic network in Fig. 2 or 4, and this was the experiment adopted here. As a consequence, by following conventional wisdom without the input of MINE, it is likely our screen would have missed the clear and unbiased identification of 295 *clock-controlled genes* in Fig. 15 or as precise an identification of the genetic network (see Table 3). The connection of the clock to ribosome biogenesis in Fig. 15B in particular is then an outcome of MINE suggesting a new direction of exploration in microarray experiments to refine our understanding of the clock mechanism.

It may at first seem surprising, or accidental, that MINE design actually helps increase the experimental sensitivity for *ccg* detection: the MINE approach, as described in Materials and Methods, optimizes the experimental sensitivity for discerning between unknown model parameter vectors Θ; it does not *per se* optimize the next experiment for detection of new molecular or gene species (such as *ccg*s) that are not explicitly included in the network model in Fig. 2. However, these two seemingly unrelated features are actually closely linked, as we will now explain.

The MINE optimization *does* tend to select experimental conditions U* which enhance the predicted response for the molecular species to be observed in the next experiment. (If the to-be-observed species were responding weakly their observation would hardly improve discrimination between different Θ). One of those to-be-observed molecular species in each of our 3 MINE cycles was the *frq* RNA. The *frq* gene, however, is co-regulated (by WCC) with all clock-controlled genes. For purposes of external perturbation response, the *frq* gene itself *is* in fact like a typical *ccg*. Thus, by choosing the MINE U* to enhance the *frq* response we are in effect also enhancing the response of all other *ccg*s, since they are subject to similar regulatory control. Hence, the MINE-directed discovery of our 295 new clock-controlled genes is *not* accidental; it is an expected and highly desirable by-product of the MINE optimization. The broader conclusion to be drawn from these considerations is that, for gene-regulatory systems, MINE optimization, in general, will improve the conditions for experimental detection of new, unknown gene species which are co-regulated with the already known species targeted by the next experiment.

There are three advances here over earlier analyses of the genome-wide logical circuitry [1], [91], [92] of fundamental processes, such as development, carbon metabolism, and the clock. One, a working kinetics model of the clock [7] gives a complete quantitative description of genetic network dynamics in Fig. 17. Two, an ensemble method was developed for identifying a genetic network with many parameters from limited data [7] with the results in Table 2. Three, a new methodology (*i.e.*, MINE design) was developed for a model-driven discovery workflow cycle (Computing Life) in profiling experiments. This new methodology resulted in the identification of most downstream *clock-controlled genes* in Fig. 15A and an unexpected connection between the clock and ribosome biogenesis in Fig. 15B.
